# Repurposing molecular imaging to map drug targets in vivo

**DOI:** 10.1038/s43856-026-01749-6

**Published:** 2026-07-13

**Authors:** Xiaoying Xu, Vincent Taelman, Pablo Jané, Eduardo Jané, Rebecca A. Dumont, Yonathan Garama, Francisco Kim, María del Val Gómez, Karim Gariani, Martin A. Walter

**Affiliations:** 1 University of Lucerne, Lucerne, Switzerland; 2 University of Geneva, Geneva, Switzerland; 3 Nuclear Medicine and Molecular Imaging Division, Geneva University Hospitals, Geneva, Switzerland; 4 Departamento de Matemática Aplicada a la Ingeniería Aeroespacial - ETSIAE, Universidad Politécnica de Madrid, Madrid, Spain; 5 Servicio de Medicina Nuclear, Hospital Universitario Ramón y Cajal, Madrid, Spain; 6 Division of Endocrinology, Diabetes, Nutrition and Patient Therapeutic Education, Geneva University Hospitals, Geneva, Switzerland; 7 St. Anna Hospital, University of Lucerne, Lucerne, Switzerland

**Keywords:** Clinical pharmacology, Predictive markers

## Abstract

**Background:**

Understanding where drug targets are expressed in the human body is essential for precision medicine, yet this information is difficult to obtain in living patients. Molecular imaging offers a non-invasive way to visualize target expression, but its application remains fragmented. We aim to develop a systematic framework to link approved drugs, their molecular targets, and existing imaging agents, with a focus on repurposing imaging strategies for clinical use.

**Methods:**

We integrate drug, target, and disease data from public databases and combine these with imaging probe annotations and transcriptomic data from more than 240,000 patient samples across multiple diseases. Co-expression analysis is used to identify candidate surrogate imaging targets for proteins that lack direct imaging agents. Statistical associations are assessed using correlation analysis with multiple testing correction.

**Results:**

Here we show that existing imaging agents can be linked to 704 therapeutic targets across 1345 diseases. Nearly half of these targets are directly imageable, while the remainder can be connected to surrogate imaging targets through co-expression. In total, more than 4000 imaging agents are identified, enabling the systematic prioritization of candidate imaging strategies across diseases.

**Conclusions:**

This study provides a framework for repurposing molecular imaging agents to visualize drug targets in vivo. The approach expands the potential of imaging to guide patient selection and treatment monitoring, and highlights opportunities to translate existing but underused imaging agents into clinically actionable biomarkers.

## Plain Language Summary

Choosing the right treatment often depends on whether a specific drug target is present in a patient’s body. This is usually checked using tissue samples, which can be invasive and only show part of the picture. Medical imaging can help by showing these targets throughout the whole body, but many imaging tools are not widely used. In this study, we brought together information on approved drugs, the targets they act on, and existing imaging methods. We also analysed gene activity data from over 240,000 patient samples to find links between drug targets and imaging markers. We found that many drug targets can already be seen in the body, either directly or through related markers. This means that existing imaging tools could potentially be reused to help doctors choose the best treatments and track how well they are working.

## Abstract (web summary)

Xu et al., develop a framework integrating drug, target, imaging, and transcriptomic data across 240,000 samples to link therapeutics with molecular imaging strategies. It connects imaging agents to 704 targets across 1,345 diseases, identifying 4,000 agents and enabling direct or surrogate imaging for most targets.

## Introduction

Precision medicine aspires to match every patient to the right therapy at the right time^1^, yet our ability to determine whether a drug target is expressed or active in vivo remains limited. Companion diagnostics, tests that identify patients most likely to benefit from a specific therapy, are central to achieving this goal^2^. The first companion diagnostic, *HercepTest™* for trastuzumab in HER2-positive breast cancer, was approved by the FDA in 1998^3^, and was followed by more than 180 subsequent assays.

Molecular imaging offers a fundamentally different approach^4^. For over eight decades, imaging has been paired with therapeutic agents, from radioiodine scans guiding thyroid treatment^5^ to modern tracers targeting somatostatin receptors or PSMA^6,7^. Unlike biopsy-based assays, imaging enables noninvasive, real-time visualization of target expression and disease burden throughout the entire body. The first FDA-approved imaging-based companion diagnostic, FerriScan for liver iron quantification^8^, illustrates the potential of a systematic framework for imaging-guided therapy selection.

Here, we introduce a comprehensive strategy that unites pharmacologic and imaging data to visualize the currently drugged portion of the *druggable genome*, referring to genes encoding targets of FDA-/EMA- or country-level-approved drugs, which is distinct from the broader ~4000-gene potentially druggable genome. We demonstrate that, in principle, approved-drug targets can be assessed either directly or indirectly through co-expressed imaging surrogates, establishing a generalizable foundation for imaging-based companion diagnostics across medicine. This approach could streamline patient selection, accelerate drug development, and extend the reach of precision medicine beyond genetic profiling toward real-time molecular phenotyping.

## Methods

### Data integration and preprocessing

Drug, target, and disease associations were compiled from Pharos, ChEMBL, DrugCentral, and the Human Disease Ontology. Imaging target information was retrieved from MICAD, PubMed, and DisGeNET. Transcriptomic datasets were downloaded from GTEx, TCGA, and GEO, and aggregated across cohorts and platforms to generate disease-specific RNA-seq profiles^9^. Sample sizes for each disease condition are provided in the corresponding figure legends. When disease-specific RNA-seq data were unavailable (e.g., for cardiac tissue), GTEx normal tissue data were used as reference controls. All datasets were standardized by gene symbol using UniProt identifiers and mapped to Ensembl gene IDs (GRCh38) to ensure cross-database consistency.

### Identification of direct and indirect druggable genes

The currently drugged portion of the *druggable genome* was defined as all genes encoding targets of FDA-/EMA- or country-level-approved drugs. The *imageable genome* included all genes with at least one reported molecular imaging agent or imaging-related publication. The intersection of these two sets defined directly imageable drug targets, while druggable genes lacking imaging agents were categorized as indirectly imageable drug targets.

### Phylogenetic and functional annotation

Protein sequences corresponding to all druggable genes were retrieved from UniProt. Phylogenetic clustering was performed using MUSCLE v5^10^, and visualized with PhyML 3.0^11^. Gene family assignments were annotated based on UniProt functional categories and HGNC nomenclature resources^12^, followed by manual curation to ensure accuracy.

### Co-expression analysis

For each target lacking an established direct imaging probe, we nominated candidate surrogate markers using co-expression analysis on normalized transcriptomic data derived from both diseased and normal tissues within the corresponding cohorts. Pairwise gene–gene associations were quantified using Pearson correlation coefficients (*r*) computed across samples within each disease context. To reduce spurious correlations and to reflect differences in sample composition and expression variance across disease areas that can be visualized in the main figures, we applied domain-specific effect-size thresholds to define meaningful co-expression: ∣*r*∣ > 0.6 with *p* < 0.05 for neurological and cardiovascular diseases, and ∣*r*∣ > 0.5 with *p* < 0.05 for oncological diseases. This reflects the typically larger sample sizes in cancer studies compared with neurological and cardiovascular cohorts, which can yield statistically significant correlations at more modest effect sizes.

For the three diseases exemplified in the paper, epilepsy (GSE140393; Supplementary Data 1), pulmonary arterial hypertension (GSE117261; Supplementary Data 2), and TCGA renal cell carcinoma (Supplementary Data 3). We expanded candidate surrogate genes by selecting gene pairs meeting the Pearson correlation coefficients |*r*| > 0.6, followed by Benjamini–Hochberg correction (adjusted *P* < 0.05). Gene pairs passing both the correlation and significance criteria were retained as significantly co-expressed and were used to construct co-expression networks and nominate candidate imageable surrogates.

### Identification of imaging tracers and structural visualization

Genes significantly co-expressed with non-imageable targets were mapped to known imaging agents using MICAD and PubMed annotations. Chemical structures of representative drugs and radiotracers were retrieved from PubChem and visualized using ChemDraw.

### Assessment of ligand activity differences

Ligands annotated with activity types *K*_i_, *K*_d_, or IC_50_ were retained for comparative analysis. For each activity type, ligand activities associated with directly and indirectly imageable druggable targets were compared using the Wilcoxon rank-sum test.

### Dataset inclusion criteria for retrospective anchoring of indirect imaging candidates

To provide a proof-of-concept for the proposed indirect imageable strategy, we performed retrospective analyses in publicly available cancer cohorts selected a priori using predefined inclusion criteria. First, datasets were required to contain: (i) documented exposure to a specific systemic therapy; (ii) an outcome endpoint suitable for association testing (e.g., progression-free survival); and (iii) matched transcriptomic data for downstream prognostic associations. Second, among eligible cohorts, we prioritized clinical context in which an imaging-based companion diagnostic would be most actionable: therapies with substantial toxicity, high treatment costs, response rates near ~50% indicating heterogeneous benefit, and the existence of alternative treatment options. These criteria were defined prior to analysis to identify clinically high-value use cases for demonstrating feasibility. Accordingly, the examples presented in the Results should be interpreted as hypothesis-prioritizing, translationally actionable showcases rather than a comprehensive evaluation across all possible datasets.

### Cox proportional hazards analysis and Cox/Kaplan–Meier visualization

We analysed an independent cohort with 174 invasive breast carcinoma patients treated with docetaxel (cBioPortal datasets^13^; study de-identifiers listed in Supplementary Data 4). Clinical annotations and endpoints were taken directly from cBioPortal clinical files. The primary endpoint was progression-free survival (PFS), defined as months from the index treatment to progression or death.

For each cohort, continuous gene expression values were log2-transformed, z-scored, and analysed using multivariable Cox proportional hazards models adjusted for tumor stage and patient age. Tumors at stages I–II were grouped as “early” and stages III–IV as “advanced.” Hazard ratios (HR) with 95% Wald confidence intervals and two-sided *P* values are reported. When multiple genes were screened, false discovery rate (FDR) correction was applied using the Benjamini–Hochberg procedure. The proportional hazards assumption was evaluated with Schoenfeld residuals (cox.zph). Kaplan–Meier curves were generated for visualization using predefined HR thresholds.

### Statistics and reproducibility

All statistical analyses were performed in R (version 4.5.1). Gene co-expression was assessed using Pearson correlation coefficients calculated across samples within each disease-specific dataset. To account for differences in cohort size and expression variability, domain-specific thresholds were applied to define meaningful associations: |*r*| > 0.6 with *P* < 0.05 for neurological and cardiovascular datasets, and |*r*| > 0.5 with *P* < 0.05 for oncological datasets. Where indicated, *P* values were adjusted for multiple testing using the Benjamini–Hochberg method, and adjusted *P* < 0.05 was considered statistically significant.

Comparisons of ligand activity distributions were performed using two-sided Wilcoxon rank-sum tests. Group-wise differences across multiple categories were assessed using one-way analysis of variance followed by Tukey’s honestly significant difference test with multiplicity-adjusted *P* values.

Survival analyses were conducted using multivariable Cox proportional hazards models with two-sided Wald tests, adjusted for tumor stage and patient age. Hazard ratios are reported with 95% confidence intervals. The proportional hazards assumption was evaluated using Schoenfeld residuals.

All analyses were performed using publicly available datasets. Sample sizes were not predetermined and were based on data availability. Cohort sizes are reported in the corresponding figure legends and “Methods” sections and range from small disease-specific datasets (*n* = 8) to large integrated transcriptomic cohorts (>240,000 samples). No randomization or blinding was applied, as the study is based on retrospective analysis of existing datasets.

No experimental replicates were performed. Reproducibility is supported by the use of standardized data processing pipelines, consistent statistical criteria, and publicly accessible source datasets.

### Ethics statement

The study involved no animal or human experimentation and therefore did not require new ethics approval. All human-derived datasets analysed in this study were obtained from publicly available, de-identified repositories, including the Cancer Genome Atlas (TCGA), the Genotype-Tissue Expression (GTEx), Therapeutically Applicable Research to Generate Effective Treatments (TARGET), and Gene Expression Omnibus (GEO), with permission for secondary use. These repositories and/or their source studies operate under established human-subjects protection, informed-consent, and data-access frameworks.

TCGA was conducted under the oversight framework of the National Cancer Institute (NCI) and the National Human Genome Research Institute (NHGRI), with all contributing studies operating under local institutional review board (IRB) approvals and informed-consent procedures.

GTEx was conducted under the oversight of the NIH Common Fund and participating institutional review boards governing Biospecimen Source Sites, with informed-consent procedures and donor authorization frameworks as described by the GTEx consortium.

TARGET-WT (Wilms Tumor) studies were performed with the approval of the Ann & Robert H. Lurie Children’s Hospital Institutional Review Board, as reported in the original TARGET WT publication, with informed consent obtained from participants or their legal guardians.

GEO datasets were generated under the ethics approvals reported in the corresponding original studies; particularly, all tissue samples from the GSE140393 study were obtained de-identified through the biorepository at the Icahn School of Medicine at Mount Sinai (ISMMS) under approved Institutional Review Board (IRB) protocols under consultation with the Ethics Committee of the Medical Board at the Mount Sinai Hospital. The GSE117261 dataset originated from the University of California, Los Angeles (UCLA) and Pulmonary Hypertension Breakthrough Initiative (PHBI) and consists of lung tissue samples gathered from multiple institutions. Ethics approval for the GSE177522 study was approved by the Clinical Research Ethics Committee of Shanxi Bethune Hospital [approval number: NO.LYLL-2025-002/PJ29].

The current study involved only secondary analysis of publicly available, de-identified data and did not involve participant contact, intervention, or access to identifiable private information. Therefore, the current study was exempt from additional IRB approval and additional informed consent requirements.

## Results

### Data pipeline

**Fig. 1:**
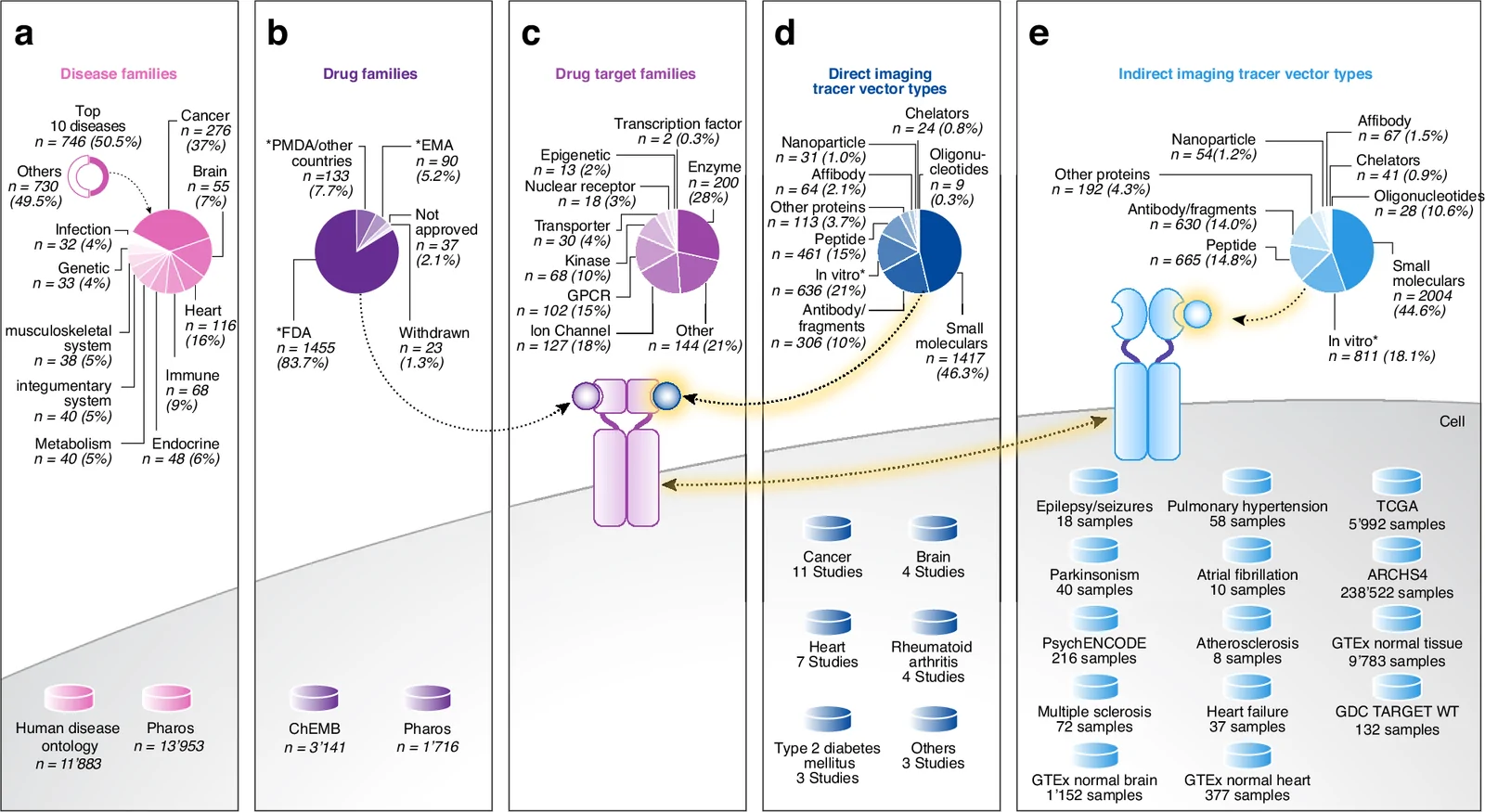
Data Pipeline. The analysis proceeds through five modules: **a** Disease. The Human Disease Ontology and Pharos datasets were integrated to define 1345 medically treatable diseases. A pie chart shows the distribution across top ten enriched diseases, including oncology, cardiology, neurology. **b** Drug. Drug data from ChEMBL and Pharos were incorporated to identify the U.S. Food and Drug Administration (FDA) and European Medicines Agency (EMA) approved drugs that have been clinically used in these 1345 diseases. **c** Drug target. Then a total of 704 drug targets were retrieved and composed the entire druggable gene dataset. **d** Direct imaging. By integrating RNAseq data from 32 studies, disease-relevant expression changes were assessed for 347 druggable genes that can be directly imaged by 3061 radiotracers. **e** Indirect imaging. Using disease-relevant RNAseq data from over 240,000 samples, 357 druggable genes can be indirectly imaged via their co-expressed genes targeted by the pool of 4492 unique radiotracers.

To systematically identify imaging-based companion diagnostics, we developed an integrative pipeline that connects medically treatable diseases, approved drugs, their molecular targets, and corresponding imaging agents (Fig. 1). Here, we define medically treatable diseases operationally as Human Disease Ontology entities that can be linked through Pharos to at least one approved therapy with an annotated molecular target. This framework bridges pharmacology and imaging by connecting the set of currently drugged targets to their imageable counterparts across human pathophysiology.

First, we combined the Human Disease Ontology^14^ and Pharos databases^15^ to identify 1345 treatable diseases (Fig. 1a) and integrated these with ChEMBL^16^ and DrugCentral^17^ to map 1700 FDA-/EMA- or country-level-approved drugs to 704 unique molecular targets (Fig. 1b, c). We then intersected these data with the Imageable Genome^18^, a concept previously defined through PubMed^19^, MICAD^20^, GEO^21^, and DisGeNET^22^, referring to genes whose protein products have reported molecular imaging probes, to determine which drug targets can already be interrogated noninvasively in vivo. This analysis identified 347 directly imageable targets corresponding to 3061 known radiotracers (Fig. 1d). While imaging ultimately measures proteins and biological processes, we use a gene-centric representation to harmonize drug–target annotation with transcriptomic resources and to support systematic discovery of imaging-enabled translational opportunities. For the remaining 357 drug targets, we used transcriptomic data from over 240,000 patient samples from GTEx^23^, TCGA^24^, and GEO^21^ to identify co-expressed imaging surrogates. Mapping these surrogates back to their associated imaging agents revealed over 4400 radiotracers, enabling the prioritization of candidate imaging strategies across the full set of drug targets within the *druggable genome* (Fig. 1e). Together, these results demonstrate that molecular imaging may provide companion diagnostics for virtually most of approved drugs targeting the human *druggable genome*. We next characterized the resulting set of 704 druggable genes to understand their structural diversity and imaging accessibility.

### The *druggable genome*

**Fig. 2:**
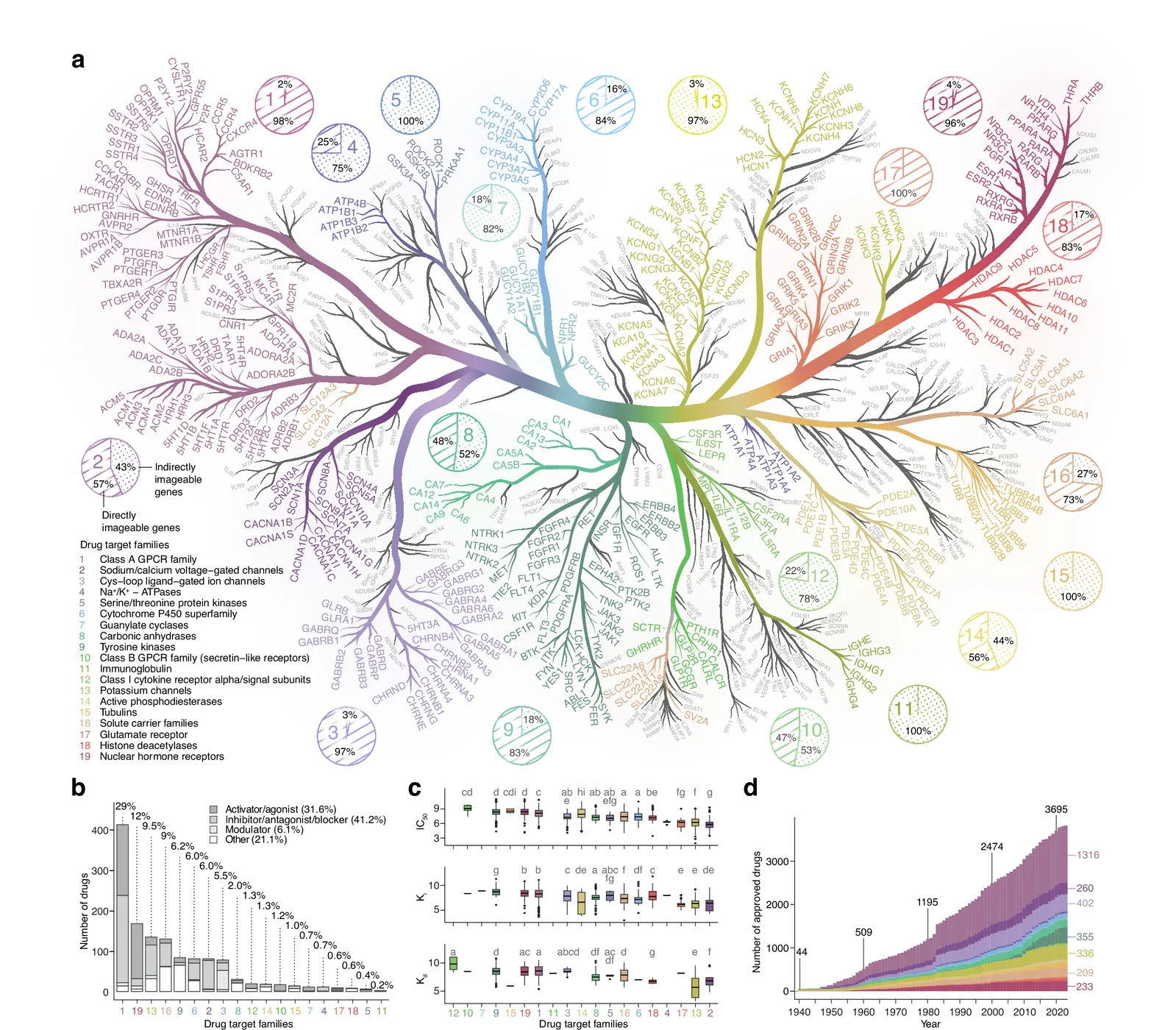
The *druggable genome.* **a** Phylogenetic tree of 704 druggable genes, manually classified into 19 major protein families or groups based on biological function and sequence similarity. **b** Distribution of approved drugs across gene groups. Drugs are categorized into four mechanistic classes. **c** Comparison of ligand binding activities among 19 major groups in tree. Statistical significance was assessed using a one-way ANOVA (*α* = 0.05, two-sided). Pairwise differences were assessed with Tukey’s honestly significant difference (HSD) test with multiplicity-adjusted *P* values; compact letter displays (CLD) indicate groups that do not differ (same letter) or do differ (different letters). Boxes represent the interquartile range (IQR), whiskers extend to 1.5× IQR, and points indicate outliers. The horizontal line indicates the mean. The *n* value represents the number of independent ligand-target activity measurements per group: active phosphodiesterases (*n* = 220 IC_50_, 30 *K*_i_); carbonic anhydrases (*n* = 188 IC_50_, 136 *K*_d_, 3107 *K*_i_); class A GPCR family (*n* = 888 IC_50_, 135 *K*_d_, 3470 *K*_i_); class B GPCR family (*n* = 8 IC_50_, 1 *K*_d_, 1 *K*_i_); Cys-loop ligand-gated ion channels (*n* = 47 IC_50_, 10 *K*_d_, 227 *K*_i_); cytochrome P450 superfamily (*n* = 400 IC_50_, 1 *K*_d_, 77 *K*_i_); glutamate receptor (*n* = 19 IC_50_, 1 *K*_d_, 50 *K*_i_); guanylate cyclases (*n* = 1 *K*_i_); histone deacetylases (*n* = 1041 IC_50_, 3 *K*_d_, 165 *K*_i_); immunoglobulin (*n* = 1 *K*_d_); Na^+^/K^+^-ATPases (*n* = 10 IC_50_, 2 *K*_i_); nuclear hormone receptors (*n* = 669 I IC_50_, 130 *K*_d_, 364 *K*_i_); potassium channels (*n* = 557 IC_50_, 16 *K*_d_, 59 *K*_i_); serine/threonine protein kinases (*n* = 12 | IC_50_, 6 *K*_d_, 8 *K*_i_); sodium/calcium voltage-gated channels (*n* = 187 IC_50_, 13 *K*_d_, 13 *K*_i_); solute carrier families (*n* = 543 IC_50_, 60 *K*_d_, 427 *K*_i_); tyrosine kinases (*n* = 1817 IC_50_, 732 *K*_d_, 138 *K*_i_). Tubulins (*n* = 23 IC_50_, 1 *K*_d_). Class I cytokine receptor alpha/signal subunits (*n* = 7 *K*_d_). **d** Total number of FDA approved drugs from year 1930 to 2023, displayed as a stacked bar chart. Each bar indicates the sum of drugs approved until that year. Color segments representing the corresponding gene groups in tree.

We first analysed the molecular diversity of the 704 currently druggable genes, with amino acid sequences retrieved from UniProt^25^, to define the structural and functional landscape of the *druggable genome*. Phylogenetic analysis identified 19 major target families, including kinases, G-protein-coupled receptors, ion channels, enzymes, and nuclear receptors (Fig. 2a and Supplementary Data 5). These families are targeted by drugs acting as inhibitors, antagonists, blockers, agonists, activators, or modulators at variable proportions (Fig. 2b and Supplementary Data 5). Drug-target affinity profiles differed markedly across families (Fig. 2c and Supplementary Data 5), and the number of targets has expanded at an average rate of 46 per year since 1930 (Fig. 2d and Supplementary Data 6).

Gene family-level analyses revealed substantial heterogeneity in imaging accessibility. Glutamate receptors, for example, are universally imageable owing to their membrane localization and extracellular binding domains, which facilitate tracer design^26^. By contrast, intracellular proteins, such as tubulins, are rarely directly imageable because their localization restricts tracer access^27^. Immunoglobulins are seldom directly imageable due to widespread expression but frequently serve as vehicles for targeted imaging agents^28^. Class B GPCRs, carbonic anhydrases, and voltage-gated ion channels occupy an intermediate position, exhibiting both direct and indirect imageability.

Together, these findings show that strategies to image the entire *druggable genome* must combine direct visualization of accessible targets with indirect imaging through co-expressed surrogates for less accessible ones.

### Direct imaging of the *druggable genome*

**Fig. 3:**
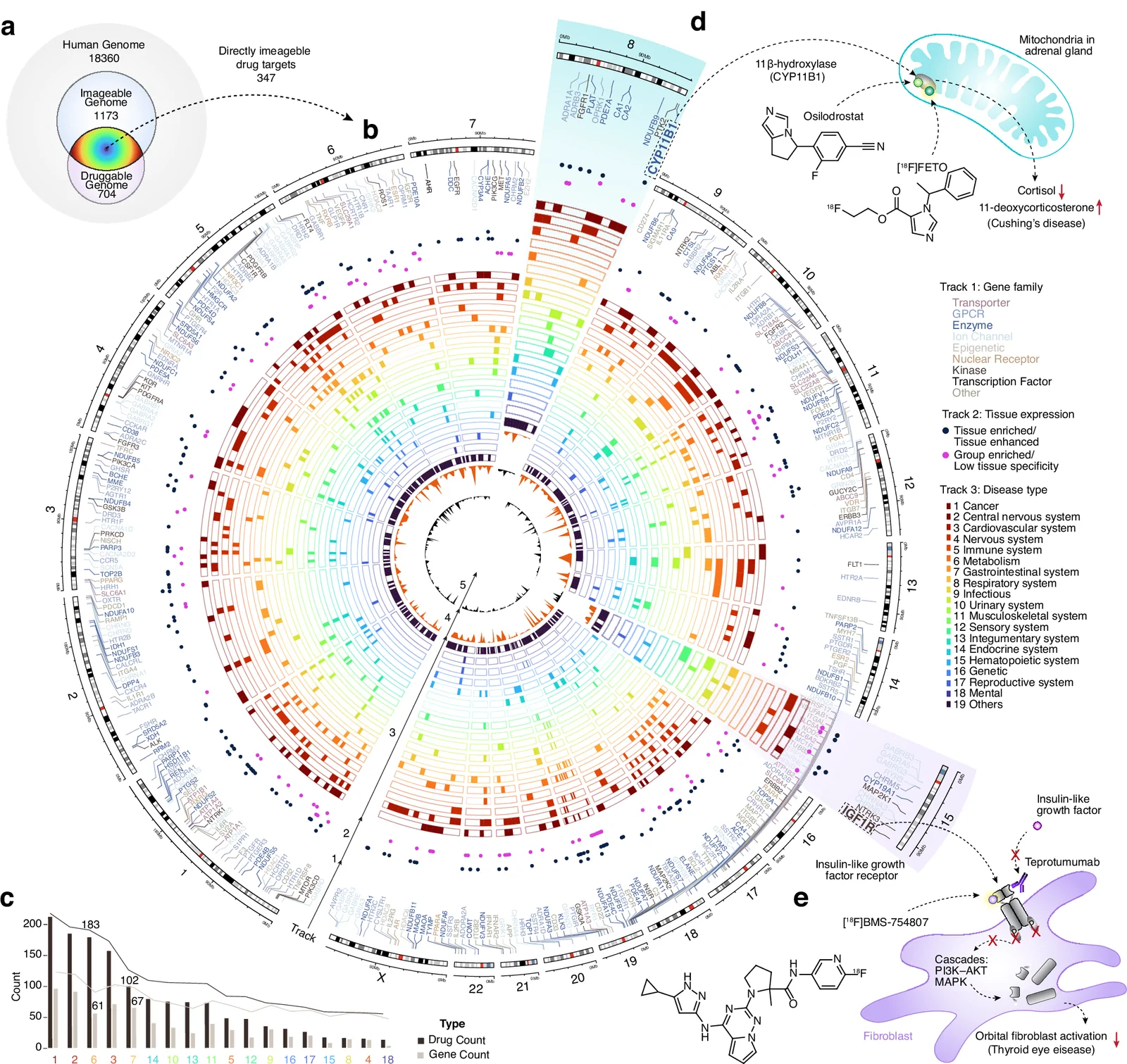
Functional landscape of 347 directly imageable druggable genes. **a** Overlap of the imageable and druggable genome within the human genome (GRCh38). The number of protein-coding genes is shown for each category. **b** Circos plot of genomic and functional annotation of 347 directly imageable druggable genes. *Track* 1: chromosomal distribution. *Track* 2: tissue-specific expression. *Track* 3**:** disease classification. *Track* 4: red histograms show the number of imaging tracers per gene. *Track* 5: black histograms show the number of approved drugs per gene. **c** Number of approved drugs and directly imageable druggable genes per disease category. **d** Osilodrostat blocks 11β-hydroxylase catalytic site in treating Cushing’s disease. The expression level of 11β-hydroxylase is visualized with imaging tracer [^18^F]FETO. **e** Teprotumumab targets insulin-like growth factor-1 receptor (IGF-1R) in thyroid eye disease. The drug binding caused IGF-1R degradation is visualized with imaging tracer [^18^F]BMS-754807. Chemical structures of drugs and imaging tracers are shown in each panel.

Of the 704 druggable genes, 347 (49.3%) are directly imageable using existing tracers (Fig. 3a, b, Supplementary Fig. 1, and Supplementary Data 7). These targets are broadly expressed across tissues (Fig. 3b, *track* 2), span diverse disease areas (Fig. 3b, *track* 3), and are frequently associated with multiple imaging tracers (Fig. 3b, *track* 4) and drugs (Fig. 3b, *track* 5), and occasionally expressed only in disease tissues (Supplementary Fig. 2). Directly imageable targets are particularly enriched in oncology (97 genes, 213 drugs), neurology (92 genes, 186 drugs), immunology (57 genes, 180 drugs), and cardiology (72 genes, 158 drugs) (Fig. 3c), all areas with pressing demand for companion diagnostics.

Several examples illustrate the translational potential of direct imaging. A strong example of how direct molecular imaging can support therapeutic evaluation comes from Alzheimer’s disease, where PET tracers are integrated into both trial enrichment and therapeutic assessment. In the phase 3 TRAILBLAZER-ALZ 2 donanemab trial^29^, participants with early symptomatic Alzheimer’s disease were selected and grouped using amyloid PET (^18^F-florbetapir or ^18^F-florbetaben) and tau PET (^18^F-flortaucipir). Donanemab treatment effect was then quantified directly with amyloid PET (^18^F-florbetapir or ^18^F-florbetaben), providing an imaging target-linked readout of amyloid plaque reduction that complements clinical endpoints (Supplementary Fig. 3). This Alzheimer’s study provides a well-established example in neurology where imaging functions as a companion biomarker for both pre-therapeutic selection and on-therapy response quantification. It aligns closely with our translational goal of directly imageable drug targets. Similarly, the 11β-hydroxylase inhibitor osilodrostat achieves responses in roughly 50% of patients with Cushing’s syndrome but carries significant toxicity risks and monthly costs above USD 11,000^30–32^. The tracer [^18^F]FETO^33^ could provide a potential basis for companion diagnostic development for osilodrostat response (Fig. 3d). Finally, teprotumumab, a monoclonal antibody targeting the insulin-like growth factor-1 receptor (IGF-1R, encoded by *IGF1R*), produces responses in ~70% of patients with Graves’ orbitopathy but at a 24-week course cost of USD 343,000^34^, and with frequent metabolic and auditory side effects^35,36^. We nominate [^18^F]BMS-754807 tracer as a candidate imaging readout for follow-up evaluation of teprotumumab response (Fig. 3e). This nomination is based on both the existence of a published tracer for [^18^F]BMS-754807 and disease-relevant molecular associations^37^. Clinical utility will require prospective imaging and pharmacodynamic validation.

Collectively, these cases demonstrate that direct imaging can bridge pharmacology and clinical decision-making by identifying existing tracers suitable for pre-therapeutic decision-making in high-cost, high-toxicity treatments with available alternatives. While nearly half of all drug targets can already be visualized directly, many remain inaccessible to current imaging agents and thus require indirect approaches based on co-expression networks.

### Indirect imaging of the *druggable genome* in neurology

Neurological disorders are biologically heterogeneous and often require long-term, costly treatments with modest average efficacy^38^. Imaging-based companion diagnostics can identify patients who harbor the relevant molecular pathology, avoiding prolonged exposure of nonresponders to ineffective or toxic therapy. This is especially valuable in central nervous system disorders, where direct tissue sampling is rarely feasible.

**Fig. 4:**
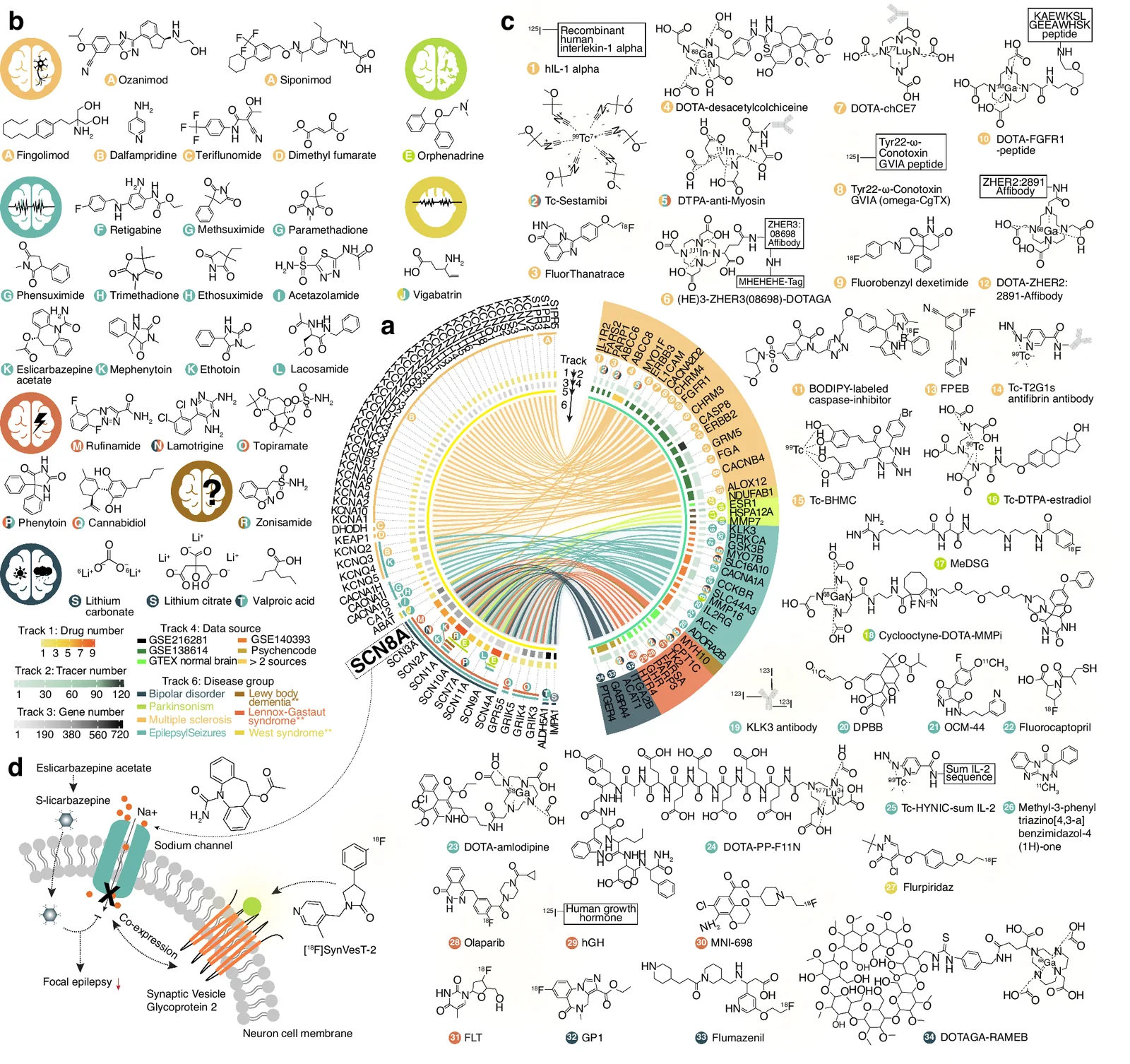
Indirect imaging of central nervous disorders. **a** Chord diagram illustrating associations between indirectly imageable druggable genes and their co-expressed, directly imageable counterparts across major human brain disorders. FDA approved drugs (left semicircle) and radiotracers (right semicircle) targeting these genes are color-coded by disease type. *Track* 1: number of FDA approved drugs targeting each indirectly imageable gene (color-coded). *Track* 2: number of radiotracers available for each co-expressed, directly imageable gene (color-coded). *Track* 3: number of co-expressed imageable genes associated with each indirectly imageable gene (color-coded). *Track* 4: RNA-seq datasets used for gene co-expression analysis. *Track* 5: genes categorized as either indirectly imageable druggable (yellow) or co-expressed directly imageable (green), associated with major brain diseases. *Track* 6: links between indirectly imageable druggable genes and their co-expressed imageable partners, color-coded by corresponding major brain disease. Sample size: bipolar disorder (*n* = 216), Parkinsonism (*n* = 40), multiple sclerosis (*n* = 72), epilepsy/seizures (*n* = 18), normal brain (GTEx; *n* = 115). **b** Chemical structures of FDA approved drugs targeting indirectly imageable genes across seven major brain diseases. **c** Representative chemical structures of radiotracers used to image selected co-expressed imageable genes. **d** An illustration of imaging target, SV2A, as a potential companion diagnostic for eslicarbazepine acetate treatment in focal epilepsy. **Due to limited disease-specific heart data, normal heart tissue samples from GTEx were used for correlation analysis. A full list of radiotracers provided in Supplementary Data 8.

To identify drug targets that are indirectly imageable through co-expression with imaging surrogates, we analysed brain-specific transcriptomic data from 1498 patients. We mapped all 29 FDA-/EMA- or country-level-approved drugs used for bipolar disorder, Parkinsonism, multiple sclerosis, epilepsy, Lewy body dementia, Lennox-Gastaut syndrome, and West syndrome (Fig. 4a and Supplementary Data 8), to their 64 molecular targets (Fig. 4b, *left semicircle*), and identified 45 co-expressed imaging targets (Fig. 4b, *right semicircle*). These corresponded to 478 unique imaging tracers that could serve as candidate companion diagnostics (Fig. 4c and Supplementary Data 8).

As an illustrative example, eslicarbazepine acetate blocks voltage-gated sodium channels (encoded by *SCN8A*), achieving responses in approximately 50–70% of patients with focal epilepsy^39^. Although relatively affordable, treatment is frequently limited by dizziness, somnolence, and breakthrough seizures, underscoring the need for biomarkers of efficacy^40–42^. *SV2A* (encoding synaptic vesicle glycoprotein 2A) and *SCN8A* both participate in neurotransmitter release and neuronal excitability control^43,44^, and are co-expressed (correlation score =  0.87, adjusted *P* = 5.19 × 10^−^^5^, *n* = 18, Fig. 4d and Supplementary Data 1). In this context, SV2A PET (e.g., [^18^F]SynVesT-2^45^), currently used primarily as a synaptic density marker, may therefore represent a practical imaging modality to evaluate synaptic integrity in this clinical context, and could be considered for future studies assessing treatment-related changes. However, whether SV2A PET can serve as a surrogate for SCN8A modulation or predict eslicarbazepine acetate response remains to be established.

### Indirect imaging of the *druggable genome* in cardiology

Cardiovascular therapy often requires balancing efficacy against adverse events, such as bleeding or hypotension^46^. Companion diagnostics could personalize treatment selection and dosing, improving safety and cost-effectiveness in a field where interindividual variability in drug metabolism is substantial.

**Fig. 5:**
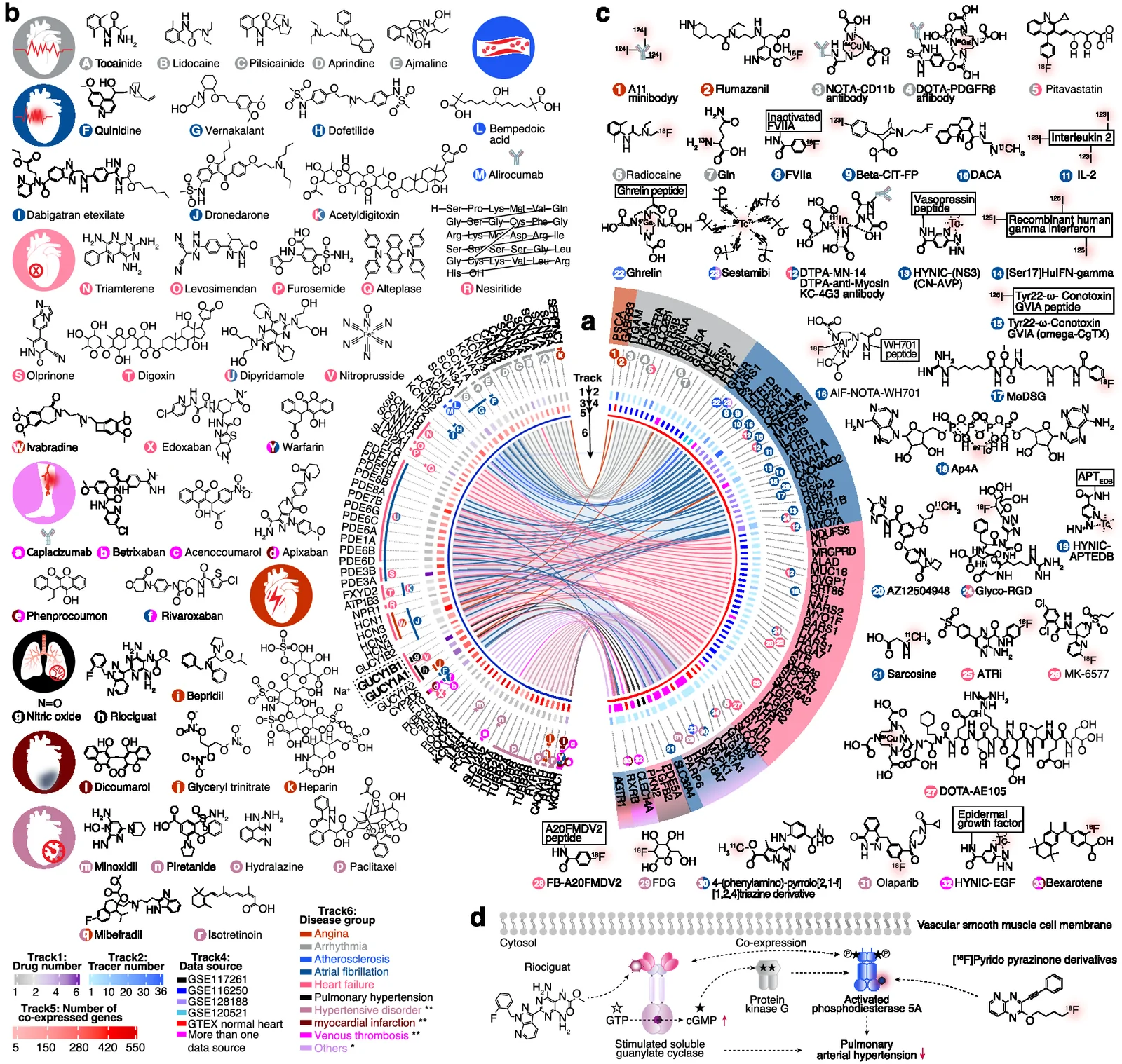
Indirect molecular imaging of major heart diseases via co-expression networks. **a** Chord diagram illustrating the relationships between indirectly imageable drug targets and their co-expressed imageable partners across major human heart diseases. Track (outer → inner): (1) number of approved drugs targeting indirectly imageable genes; (2) number of radiotracers imaging each co-expressed gene; (3) number of co-expressed imageable genes associated with each indirectly imageable drug target; (4) RNA-seq datasets used for co-expression analysis; (5) classification of genes as indirectly imageable drug targets (blue) or co-expressed imageable genes (red), implicated in major heart diseases; (6) links between indirectly imageable drug targets and their co-expressed imageable partners, color-coded by disease type. Sample sizes: angina (*n* = 13), arrhythmia (*n* = 19), atherosclerosis (*n* = 8), atrial fibrillation (*n* = 10), heart failure (*n* = 37), pulmonary hypertension (*n* = 58), and normal heart tissue (GTEx; *n* = 377). **b** Chemical structures of FDA approved drugs targeting indirectly imageable genes implicated in nine major human heart diseases. **c** Representative chemical structures of radiotracers used to image selected co-expressed imageable genes (correlation score >0.82, *P* < 0.01). **d** An illustration of imaging targets PDE5A as a potential companion diagnostic for riociguat treatment in pulmonary arterial hypertension. *Drugs targeting indirectly imageable drug targets associated with other heart diseases are provided in Supplementary Data 7. **Due to the lack of disease-specific transcriptomic data, normal heart tissue samples (GTEx) were used for correlation analysis. A full list of radiotracers provided in Supplementary Data 9.

Using cardiovascular-specific transcriptomic datasets from 505 patients, we mapped 89 approved drugs indicated for angina, arrhythmia, atherosclerosis, atrial fibrillation, heart failure, hypertension, myocardial infarction, pulmonary hypertension, and venous thrombosis (Fig. 5a and Supplementary Data 7) to 72 molecular drug targets (Fig. 5b, *left semicircle*). We then identified 75 co-expressed imaging targets (Fig. 5b, *right semicircle*) and 339 corresponding imaging tracers that could serve as potential companion diagnostics (Fig. 5c and Supplementary Data 9).

Riociguat, a stimulator of soluble guanylate cyclase encoded by *GUCY1A1* and *GUCY1B1*, enhances responsiveness to endogenous nitric oxide by increasing cyclic guanosine monophosphate (cGMP) production^47^. Although effective in roughly half of patients with pulmonary arterial hypertension^48^, annual treatment costs exceed USD 46,000^49^, and adverse effects, such as hypotension and syncope, are frequent^50,51^. Phosphodiesterase 5A (encoded by *PDE5A*) and soluble guanylate cyclase (α1 and β1 units encoded by *GUCY1A1 and GUCY1B1*) act in a feedback loop within the nitric oxide–cGMP signaling pathway, where GUCY1A1 generates and PDE5A degrades the second messenger cGMP, thereby jointly regulating signal duration and intensity^52^. *PDE5A* is co-expressed with *GUCY1A1* (correlation score = 0.67, adjusted *P* = 8.16 × 10^−^^7^, *n* = 58) and *GUCY1B1* (correlation score = 0.65, adjusted *P* = 1.41 × 10^−^^6^, *n* = 58; Fig. 5d and Supplementary Data 2). Thus, fluorine-18-labelled pyridopyrazinone derivatives, which target PDE5A, could serve as noninvasive imaging biomarkers for patient selection and treatment monitoring during riociguat therapy.

### Indirect imaging of the *druggable genome* in **oncology**

Cancer therapy is marked by high biological heterogeneity, significant toxicity, and extreme treatment costs. Imaging-based companion diagnostics can identify responsive molecular subgroups, prevent futile exposure to toxic drugs, and guide adaptive treatment strategies^53^.

**Fig. 6:**
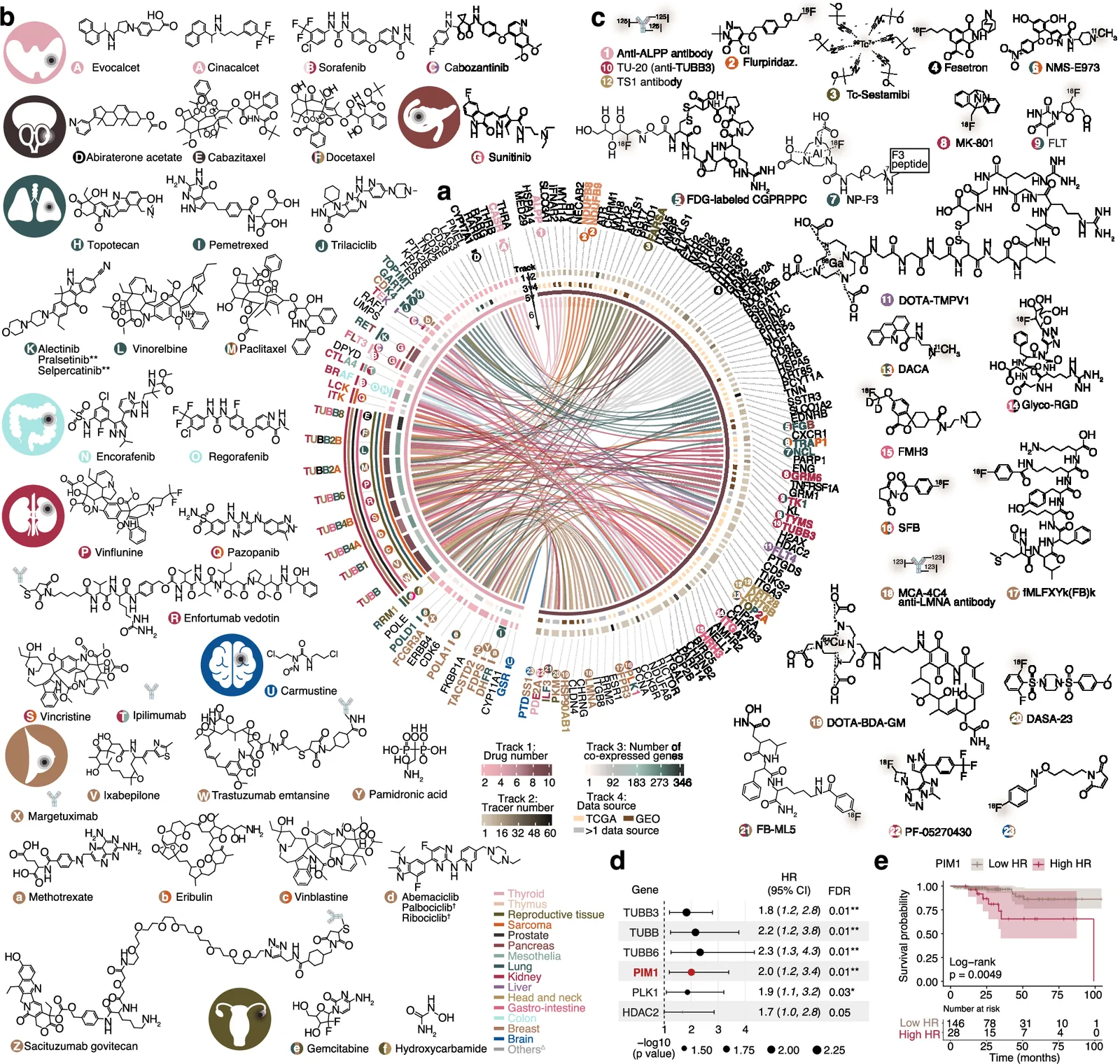
Indirect molecular imaging of human cancers via co-expression networks. **a** Chord diagram linking indirectly imageable drug targets to co-expressed imageable genes across major cancers. Tracks (outer → inner): (1) number of FDA approved drugs per target gene; (2) number of radiotracers per co-expressed imageable gene; (3) number of co-expressed imageable genes associated with each drug target; (4) RNA-seq datasets used for co-expression; (5) gene class (indirectly imageable, dust pink; co-expressed imageable, dark mauve); (6) co-expression links colored by cancer type. Sample sizes (TCGA; GEO unless noted): thyroid (502; 234), thymus (GSE177522, 19), reproductive tissue (2815), sarcoma (259; 2250), prostate (498), pancreas (177; 961), mesothelioma (86), lung (502; 1780), kidney (533; 349; TARGET WT, 132), liver (371), head and neck (500; 2250), gastrointestinal (375), colon (2227), breast (1102; 5441), and brain (2750). **b** Representative approved drugs structures targeting indirectly imageable drug targets implicated in major cancers. **c** Representative radiotracer structures for selected co-expressed imageable genes (*r* > 0.7; *P* < 0.05). **d** Two-sided Wald tests from stage- and age-adjusted Cox proportional hazards models are summarized as a forest plot for drug target genes and their co-expressed genes (*n* = 1102 biologically independent TCGA breast invasive carcinoma patients, co-expression filter *r* > 0.6 and *P* < 1.0e-100). Points show hazard ratios (HRs) and error bars indicate 95% Wald confidence intervals (CIs). FDR-adjusted *P* values were computed using the Benjamini–Hochberg procedure across model terms. **e** Progression-free survival (PFS) Kaplan–M**e**ier curves comparing high-risk (HR > 2.0) versus low-risk (HR ≤ 2.0) PIM1 groups, defined by the PIM1-specific Cox linear predictor from an age- and stage-adjusted model. *P* values are from two-sided log-rank tests. Shaded areas indicate 95% confidence intervals (CIs). ^∆^Drugs involved in other cancer types are provided in Supplementary Data 7. ^†^Compound chemical structures are detailed in Supplementary Fig. 4a. A full list of radiotracers is provided in Supplementary Data 10.

Using cancer-specific transcriptomic data from 244,658 patients, we mapped 49 approved anticancer drugs across 66 tumor types (Fig. 6a and Supplementary Data 7), to 48 molecular targets (Fig. 6b, *left semicircle*). We then identified 99 co-expressed imaging targets (Fig. 6b, *right semicircle*), corresponding to 602 candidate imaging tracers (Fig. 6c and Supplementary Data 10).

The following two retrospective examples are presented as a priori–selected, clinically high-impact showcases of the indirect imageable strategy (high toxicity and cost, heterogeneous response, and actionable alternatives), and are not intended as an exhaustive evaluation across all datasets. For example, lenvatinib is a multikinase inhibitor that blocks the RET proto-oncogene (encoded by *RET*), achieving response rates of 71% in advanced renal cell carcinoma^54,55^. Treatment costs exceed USD 150,000 per year, and side effects, such as hypertension, fatigue, and proteinuria, are common^56–58^. The PET tracer [^18^F]NFEP targets neuronal nicotinic acetylcholine receptors that are co-expressed with *RET* in the TCGA-KIRC cohort (correlation score = 0.67, adjusted *P* = 4.0 × 10^−^^69^, *n* = 533 patients, Supplementary Fig. 4b and Supplementary Data 3), suggesting a testable lead imaging candidate for companion diagnostic development in RET-driven cancers.

To empirically anchor the indirect imaging strategy with therapy-linked clinical outcome data, we validated one predicted indirect imaging pair in a clinical cohort of 174 patients with invasive breast carcinoma receiving docetaxel therapy. Docetaxel targets β-tubulin (TUBB6 protein; encoded by *TUBB6*), a non-imageable intracellular protein, but its transcriptomic expression is tightly correlated with that of the proto-oncogene *PIM1* (*r* = 0.68, adjusted *P* < 10^−4^, Supplementary Fig. 5). *TUBB6* and *PIM1* converge on pathways of cell proliferation and oncogenesis, PIM1 as a proto-oncogene promoting growth, and TUBB6 through altered microtubule dynamics that facilitate cancer progression^59,60^.

In this cohort, expression of both *TUBB6* and *PIM1* independently predicts progression-free survival (*PIM1*: HR = 2.0, 95% CI 1.2–3.4, adjusted *P* = 0.01; *TUBB6*: HR = 2.3, 95% CI 1.3–4.3, adjusted *P* = 0.01, Fig. 6d, e). Together with their co-expression, these retrospective associations support PIM1 as a plausible, testable imaging surrogate for a docetaxel-relevant biological state. Importantly, PIM1 can be visualized using the PET radiotracer [^11^C]CX-6258^61^, It provides a concrete translational route to evaluate this hypothesis in vivo. However, demonstrating clinical utility would require prospective studies showing that baseline or on-treatment PIM1 tracer uptake (and/or pharmacodynamic changes in uptake) correlates with docetaxel sensitivity or resistance and improves outcome prediction beyond existing clinical and molecular factors. Accordingly, this example is presented to prioritize a candidate tracer–target strategy and to outline a feasible path for future imaging-based validation.

Overall, these results indicate that indirect imaging may substantially extend molecular phenotyping across therapeutic domains, including settings where direct target imaging is not currently feasible.

## Discussion

The present study provides a comprehensive framework to visualize the currently drugged portion of the *druggable genome* in vivo, establishing a direct link between pharmacology and molecular imaging. In this study, we define the currently drugged portion of the *druggable genome* as genes encoding targets of FDA-/EMA- or country-level approved drugs, prioritizing immediate translational relevance. This clinically grounded approach enables direct linkage between existing therapeutics and imaging strategies for patient stratification and treatment monitoring. In contrast, broader definitions (e.g., OpenTargets) include predicted or investigational targets that expand the target landscape but fall outside the scope of this work. Our framework is therefore complementary, providing a focused, actionable subset that can be extended in future studies to incorporate emerging targets. Importantly, this work systematically maps the FDA-/EMA- or country-level-approved *druggable genome* to currently available molecular imaging tracers through both direct target imaging and indirect imaging via co-expressed imageable genes in disease-relevant tissues. The resulting drug–target–tracer relationships, including family classifications, direct imageable targets, and disease-specific indirect imaging networks across neurology, cardiology, and oncology, are provided in Supplementary Data 5–10. These curated source tables establish a reusable translational resource linking pharmacology, transcriptomics, and molecular imaging across clinically actionable disease contexts.

By integrating genomic, transcriptomic, and imaging datasets covering more than 1600 approved drugs and 700 therapeutic targets, we demonstrate that many approved-drug targets can, in principle, be visualized either directly or prioritized for indirect imaging through co-expressed surrogates. This conclusion is supported by empirical validation in docetaxel-treated breast carcinoma patients and represents a conceptual and technical advance that extends the reach of precision medicine from molecular discovery to noninvasive patient stratification. Beyond its immediate applications in oncology, neurology, and cardiology, the framework defines a broadly applicable platform for imaging-based companion diagnostics, offering a scalable roadmap for data-driven drug development.

Because the tracer candidates curated here are derived from peer-reviewed published studies, many of the practical parameters relevant to translation (e.g., radionuclide, half-life, known radiometabolites, plasma free fraction, and evidence of barrier penetration) are already established in the primary literature and can be evaluated during indication-specific prioritization. Accordingly, we use “imageable” to mean that a published imaging probe exists for a given target, while recognizing that whether it can be used clinically still depends on the specific setting and on the tracer’s real-world performance and quantification.

The ability to visualize drug target expression in the living human body has far-reaching clinical implications that extend across all major disease areas. In oncology, where therapeutic toxicity and cost are high, imaging companion diagnostics can identify responders, avoid futile exposure, and monitor emerging resistance, all in real time. In cardiology, imaging can refine risk-benefit assessments for potent or expensive interventions by mapping molecular pathways of vascular remodeling, inflammation, and myocardial stress. In neurology, where biopsy access is limited and diseases progress slowly, imaging enables noninvasive evaluation of therapeutic targets directly within the brain, improving trial efficiency and patient selection. Beyond these domains, the same framework can support treatment guidance in immunology, endocrinology, and infectious disease, wherever knowledge of target expression can inform therapeutic decisions^2^.

Across disciplines, three advantages emerge. First, imaging introduces *functional precision*: rather than relying solely on genotype, clinicians can stratify patients by real-time molecular phenotype. Second, imaging provides *spatial and temporal resolution*: it captures the heterogeneity of target expression across organs and over time, something that no biopsy-based test can achieve. Third, imaging delivers *actionable feedback*: the same modality that predicts response can also quantify target engagement and guide therapy adjustment during treatment. Fourth, imaging provides a reproducible map of normal-organ biodistribution that quantifies physiologic uptake across healthy tissues. Such reference data can be directly compared with tracer uptake in target lesions (e.g., tumor-to-organ ratios, residence times) to *anticipate* and *mitigate toxicity* on an individual-patient level. Together, these capabilities position imaging-based companion diagnostics as a unifying clinical tool, one that links disease biology, therapeutic mechanism, and patient outcome in a single noninvasive readout.

Drug development remains slow, costly, and failure-prone, largely due to uncertainty about target engagement and patient selection^62–64^. Imaging the *druggable genome* offers a framework to make drug development more predictive, more efficient, and more mechanistically informed.

First, *target validation* could be accelerated. Imaging enables direct visualization of target expression in relevant tissues and patient populations before expensive efficacy trials begin, allowing early termination of projects with insufficient in vivo target engagement. Second, *early-phase trials* could become more informative. PET-based readouts of drug–target interaction provide quantitative biomarkers of pharmacodynamics and can replace or complement invasive biopsies. Beyond tracer availability, recent advances in total-body PET, using long axial field-of-view scanners with much higher detection sensitivity, can make many molecular probes more clinically practical. Total-body PET can collect dynamic data across the whole body at the same time, enabling system-wide kinetic analysis and quantitative measurement of target signal across organs and lesions in a single scan, often with shorter scan times and/or lower injected activity. Importantly, the sensitivity gain can also help bring “difficult” tracers closer to routine use by improving signal-to-noise and reducing the burden of low-count imaging. This is particularly valuable for (i) mapping heterogeneous target expression across all disease sites, (ii) separating true target binding from confounders, such as blood pool activity and radiometabolites through improved kinetic characterization, and (iii) longitudinal pharmacodynamic monitoring of treatment effects across the full disease burden. In the context of our atlas, total-body PET can help narrow the gap between “reported” and “clinically deployable” imaging strategies by making whole-body, quantitative evaluation of candidate tracers more feasible in practice.

Early-phase imaging can also profile biodistribution and target-to-healthy-organ uptake to identify exposure patterns predictive of toxicity before clinical scale-up. Such imaging biomarkers can support adaptive trial designs and early go/no-go decisions, reducing both sample size and development cost. Our validation in docetaxel-treated breast carcinoma illustrates this principle, showing how transcriptional co-expression can predict imaging surrogates that stratify real-world treatment outcomes. Third, *patient stratification* would be refined. Imaging can prospectively identify biomarker-positive subgroups, improving statistical power and effect size estimation. Fourth, *mechanistic understanding* would deepen: by integrating imaging with transcriptomic and pharmacologic data, researchers can map compensatory pathways and off-target effects, guiding rational combination therapies. Fifth, *drug repurposing* could be systematized. Our atlas reveals overlap between imaging and drug targets across disease families, suggesting that existing imaging agents can accelerate translation of emerging drugs for new indications. Finally, *regulatory* and *reimbursement pathways* may benefit. Imaging biomarkers are increasingly accepted as surrogate endpoints or companion diagnostics; integrating them from the outset can shorten regulatory review and support payer evidence requirements.

Overall, imaging the *druggable genome* could help reposition molecular imaging from a diagnostic adjunct toward a central tool in drug discovery, linking the theranostic genome, the imageable genome, and the clinical phenotype in one iterative cycle of translation.

For molecular imaging itself, imaging the *druggable genome* represents both a validation of its founding vision and a major opportunity for renewal. Nuclear medicine was originally conceived to visualize molecular processes underlying disease and therapy; this work extends that mission to the entire spectrum of druggable targets.

First, *increased clinical relevance* can be expected. By anchoring imaging directly to therapeutic decision-making, radiotracers become actionable biomarkers rather than purely diagnostic tools. This might increase clinical demand, imaging volume, and reimbursement relevance, moving molecular imaging closer to the centre of clinical oncology, neurology, and cardiology. Second, *standardization*and *regulatory recognition* will improve. Imaging-based companion diagnostics, once systematically defined, can follow established co-approval pathways like IVD-based companion diagnostics, raising the field’s visibility in drug regulation and reimbursement frameworks. Third, *scientific integration* will deepen. Linking the imageable, druggable, and theranostic genomes aligns radiochemistry, bioinformatics, and pharmacology into a unified data ecosystem that continuously generates new tracer–target pairs. Fourth, *training* and *collaboration* will evolve. Radiologists and nuclear medicine specialists can contribute to early-stage drug development, bridging preclinical imaging, regulatory science, and therapeutic trials, an expanded role that redefines the specialty’s position in precision medicine. Finally, *societal* and *economic impact* will grow. Imaging-based companion diagnostics can make high-cost therapies more efficient, thus supporting the long-term sustainability of personalized healthcare^65–67^.

In essence, imaging the *druggable genome* highlights how molecular imaging could evolve from a niche diagnostic modality into a central technology supporting future therapeutics. It allows the field to fulfil its original promise, to see and quantify the molecular determinants of treatment response throughout the human body.

Genomic biomarkers are powerful for identifying oncogenic drivers and DNA repair defects, yet they often provide an incomplete view of functional drug susceptibility in individual patients. In practice, a genomic alteration (e.g., BRCA mutation or HRD status) does not guarantee uniform pathway dependence across all lesions, and it cannot capture key determinants of response, such as spatial heterogeneity, temporal adaptation under therapy, target abundance at the protein level, and drug delivery/target engagement in vivo. Functional precision—i.e., matching therapy to the current molecular state of disease—therefore benefits from readouts that can survey the entire tumor burden and its heterogeneity in the living patient. Molecular imaging is uniquely positioned to provide such whole-body, quantitative measurements repeatedly over time, enabling patient selection, early response assessment, and detection of resistant subclones emerging during treatment.

A clinically relevant illustration is the use of PARP inhibitors (PARPi) in tumors selected by BRCA mutation and/or HRD. Although these genomic criteria enrich for PARPi benefit, a substantial fraction of genomically selected patients still derive limited response, highlighting the need for additional biomarkers that better reflect functional target biology at the time of treatment. PARP1-targeted PET using radiolabeled PARP inhibitors has emerged as a compelling strategy to address this gap by noninvasively measuring PARP-related biology across lesions. For example, [^18^F]FluorThanatrace ([^18^F]FTT) has been evaluated as a functional imaging biomarker in multiple tumor contexts^68^, including studies linking baseline tracer uptake and/or changes on therapy to clinically relevant measures of PARP-imaging probe [carbonyl-^11^C]DPQ sensitivity or response^69^. In advanced prostate cancer, a pilot study using PARP1 imaging further supports feasibility of imaging PARP biology in patients and motivates prospective evaluation for treatment guidance^70^. Additional radiolabeled PARP inhibitors, [^18^F]PARPi^71,72^ and ^18^F-olaparib, have also demonstrated the ability to image and quantify PARP1 expression in vivo, providing a broader toolkit for clinical translation and comparative optimization across indications. Collectively, these studies exemplify how molecular imaging can extend beyond static genomic classification by measuring target presence and heterogeneity in vivo, thereby refining patient selection and enabling response assessment in a way that is difficult to achieve with tissue genomics alone.

In the context of the present work, PARP PET provides a concrete proof-of-principle for the broader premise of our atlas: that linking the landscape of available (including underused) tracers to clinical decision points can reveal actionable opportunities for imaging-enabled precision medicine. In future implementations, the same logic can be applied to other drug classes where genomic biomarkers are imperfect proxies for functional dependency, provided that tracer quantification performance is established and prospective validation is pursued in the intended clinical setting.

Surrogate nomination is based on mRNA co-expression, which is widely available but does not ensure concordant protein abundance because of post-transcriptional regulation, translation, and degradation dynamics, alternative splicing, and cell-type composition effects. Moreover, PET/SPECT signals are not determined by expression alone and can be influenced by delivery and permeability, nonspecific binding, radio-metabolites, barrier transport, target accessibility/compartmentalization, internalization, and endogenous or drug occupancy. Co-expression links should therefore be interpreted as prioritized, testable hypotheses, and tracer–target applications require tracer-specific quantitative validation in the intended clinical setting.

## Conclusion

By bridging pharmacology, transcriptomics, and molecular imaging, this study provides a comprehensive blueprint for imaging the *druggable genome*. Beyond a data resource, it outlines a translational vision: in the clinic, to make therapy selection more precise; in drug development, to make discovery more efficient; and in molecular imaging, to make the discipline indispensable to modern therapeutics. Together, these implications position imaging of the *druggable genome* as a practical bridge between diagnostic imaging and therapeutic development, aligning both within a shared framework of precision medicine.

## Supplementary information

Transparent Peer Review File

Supplementary Information

Description of Additional Supplementary Data

Supplementary Data 1

Supplementary Data 2

Supplementary Data 3

Supplementary Data 4

Supplementary Data 5

Supplementary Data 6

Supplementary Data 7

Supplementary Data 8

Supplementary Data 9

Supplementary Data 10

Supplementary Data 11

Supplementary Data 12

Supplementary Data 13

## Data Availability

All datasets analysed in this study are publicly available. Drug, target, disease, and ligand activity were retrieved from Pharos, ChEMBL, DrugCentral, and the Human Disease Ontology. Imaging target information was obtained from the Molecular Imaging and Contrast Agent Database (MICAD), PubMed, and DisGeNET. Drug approval year information was sourced from Drugs@FDA. Transcriptomic data from normal tissues were sourced from the Genotype-Tissue Expression (GTEx) project (https://gtexportal.org). The oncology results published here are in part based upon data generated by The Cancer Genome Atlas (TCGA) Research Network: https://www.cancer.gov/tcga, the Therapeutically Applicable Research to Generate Effective Treatments (https://www.cancer.gov/ccg/research/genome-sequencing/target) initiative, phs000471. The data used for this analysis are available at the Genomic Data Commons (https://portal.gdc.cancer.gov). Disease-specific transcriptomic datasets were sourced from the following datasets: TCGA cohorts including TCGA-THCA, TCGA-PAAD, TCGA-MESO, TCGA-SARC, TCGA-PRAD, TCGA-LUSC, TCGA-LUAD, TCGA-KIRC, TCGA-KICH, TCGA-LIHC, TCGA- GBM, TCGA-COAD, TCGA-STAD, TCGA-OV; the ARCHS4 data repository; Gene Expression Omnibus (GEO; https://www.ncbi.nlm.nih.gov/geo/): including GSE140393 (epilepsy), GSE117261 (pulmonary arterial hypertension), and GSE177522 (thymus); and the Therapeutically Applicable Research to Generate Effective Treatments (TARGET) program. TARGET-WT (Wilms Tumor) dataset. National Cancer Institute. Available at: https://portal.gdc.cancer.gov/projects/TARGET-WT. For retrospective survival analyses, genomic and clinical data from docetaxel-treated Breast Invasive Carcinoma (TCGA PanCancer Atlas) cohort available through cBioPortal. Data are provided in Supplementary Data 4 to ensure full reproducibility. Radiotracer information was compiled from the Molecular Imaging and Contrast Agent Database (MICAD) and peer-reviewed literature. Gene co-expression matrices and full list of drug–target–tracer mappings generated during this study are available from the corresponding author upon reasonable request. The Source data for Fig. 2b–d is in Supplementary Data 11. The Source data for Fig. 3b, c is in Supplementary Data 12. The Source data for Fig. 6d, e is in Supplementary Data 13.

## Code availability

The code used for data integration and analysis is available from the corresponding author upon reasonable request. Scripts for database integration and figure generation were written in R (version 4.5.1) using open-source packages as listed in the “Methods.”
