# Supplementary material for: Repurposing molecular imaging to map drug targets in vivo
Source: Commun Med (Lond). 2026 Jul 13;6:579. doi: 10.1038/s43856-026-01749-6 (PMC13745577; doi:10.1038/s43856-026-01749-6)
Supplement: Supplementary file 1 [file 43856_2026_1749_MOESM1_ESM.pdf]

nature portfolio

Peer Review File

Repurposing molecular imaging to map drug targets in vivo

Corresponding Author: Professor Martin Walter

This file contains all reviewer reports in order by version, followed by all author rebuttals in order by version.

Version 0:

Reviewer comments:

Reviewer #1

(Remarks to the Author)
The revision largely addressed my comments.

Reviewer #2

(Remarks to the Author)
The authors have done a good job in responding to the comments raised in the previous review of this manuscript except for one point. This reviewer does not agree with the authors that [18F]6h has ideal properties for quantifying S1PR1 in vivo. The washout kinetics are very slow, and the effect of the blocking agent was minimal. This indicates that the tracer has a high level of nonspecific binding and will not likely have the sensitivity needed to quantify S1PR1 in vivo. It is unclear why the authors continue to talk about this target and imaging approach when there are many other examples they could use to demonstrate their point. A review article such as this should not present data on unvalidated PET radiotracers.

Reviewer #5

(Remarks to the Author)
I have been asked to review the revised manuscript and have not seen the previous version so apologies if some of my comments may have been made before.

1. The title seems to be far to general. This is not about the total druggable genome, but more about the repurposing part. This should be made clear from the start to prevent unnecessary confusion.

2. Why did the authors come up with their own definition of the druggable genome using bespoke linkages and how does this compare against relatively canonical definitions provided by others such as opentargets.org. Not suggestion to re-do everything using opentargets definition, but simply compare whether the identify different targets of approved compounds.

3. Despite the authors claiming to have developed a resource nothing is made publicly available and they simply ask people to contact the lead author. Possibly I am mistaken and everything is actually shared? Otherwise what is the point of the paper?

4. I am glad to see the authors have taken on the comments from reviewer's 3 and 4 - the response seems fine. next time please copy the changed section verbatim in the rebuttal letter, makes it easier to say yes.

Version 1:

Reviewer comments:

Reviewer #5

(Remarks to the Author)
No further comments!

Open Access This Peer Review File is licensed under a Creative Commons Attribution 4.0 International License, which permits use, sharing, adaptation, distribution and reproduction in any medium or format, as long as you give appropriate credit to the original author(s) and the source, provide a link to the Creative Commons license, and indicate if changes were made.
In cases where reviewers are anonymous, credit should be given to 'Anonymous Referee' and the source.
The images or other third party material in this Peer Review File are included in the article's Creative Commons license, unless indicated otherwise in a credit line to the material. If material is not included in the article's Creative Commons license and your intended use is not permitted by statutory regulation or exceeds the permitted use, you will need to obtain permission directly from the copyright holder.
To view a copy of this license, visit https://creativecommons.org/licenses/by/4.0/

Point-by-point responses

Reviewer #1 (Remarks to the Author):

The revision largely addressed my comments.

Response:

We thank the reviewer for these important points, which have helped improve our work.

Reviewer #2

The authors have done a good job in responding to the comments raised in the previous review of this manuscript except for one point. This reviewer does not agree with the authors that [18F]6h has ideal properties for quantifying S1PR1 in vivo. The washout kinetics are very slow, and the effect of the blocking agent was minimal. This indicates that the tracer has a high level of nonspecific binding and will not likely have the sensitivity needed to quantify S1PR1 in vivo. It is unclear why the authors continue to talk about this target and imaging approach when there are many other examples they could use to demonstrate their point. A review article such as this should not present data on unvalidated PET radiotracers.

Response:

We thank the reviewer for highlighting this issue. We have now removed the [18F]6h example from the revised manuscript and from the Figure 3. Please check the revised paragraph "DIRECT IMAGING OF THE REPURPOSED *DRUGGABLE GENOME*", as well as the revised Figure 3:

DIRECT IMAGING OF THE *DRUGGABLE GENOME*

Of the 704 druggable genes, 347 (49.3%) are directly imageable using existing tracers (**Fig. 3a, b**; **Supplementary Fig. 1, Supplementary Data 3**). These targets are broadly expressed across tissues (**Fig. 3b**, *track 2*), span diverse disease areas (**Fig. 3b**, *track 3*), and are frequently associated with multiple imaging tracers (**Fig. 3b**, *track 4*) and drugs (**Fig. 3b**, *track 5*), and occasionally expressed only in disease tissues (**Supplementary Fig. 2**). Directly imageable targets are particularly enriched in oncology (97 genes, 213 drugs), neurology (92 genes, 186 drugs), immunology (57 genes, 180 drugs), and cardiology (72 genes, 158 drugs) (**Fig. 3c**), all areas with pressing demand for companion diagnostics.

Several examples illustrate the translational potential of direct imaging. A strong example of how direct molecular imaging can support therapeutic evaluation comes from Alzheimer's disease, where PET tracers are integrated into both trial enrichment and therapeutic assessment. In the phase 3 TRAILBLAZER-ALZ 2 donanemab trial [1], participants with early symptomatic Alzheimer's disease were selected and grouped using amyloid PET (^{18}F-florbetapir or ^{18}F-florbetaben) and tau PET (^{18}F-flortaucipir). Donanemab treatment effect was then quantified directly with amyloid PET (^{18}F-florbetapir or ^{18}F-florbetaben), providing an imaging target-linked readout of amyloid plaque reduction that complements clinical endpoints (**Supplementary Fig. 3**). This Alzheimer's study provides a well-established example in neurology where imaging functions as a companion biomarker for both pre-therapeutic selection and on-therapy response quantification. It aligns closely with our translational goal of directly imageable drug targets. Similarly, the 11β-hydroxylase inhibitor osilodrostat achieves responses in

roughly 50% of patients with Cushing's syndrome but carries significant toxicity risks and monthly costs above USD 11,000 [2,3,4]. The tracer [^{18}F]FETO [5] could provide a potential basis for companion diagnostic development for osilodrostat response (**Fig. 3d**). Finally, teprotumumab, a monoclonal antibody targeting the insulin-like growth factor-1 receptor (IGF-1R, encoded by *IGF1R*), produces responses in ~70% of patients with Graves' orbitopathy but at a 24-week course cost of USD 343,000 [6], and with frequent metabolic and auditory side effects [7,8]. We nominate [^{18}F]BMS-754807 tracer as a candidate imaging readout for follow-up evaluation of teprotumumab response (**Fig. 3e**). This nomination is based on both the existence of a published tracer for [^{18}F]BMS-754807 and disease-relevant molecular associations [9]. Clinical utility will require prospective imaging and pharmacodynamic validation.

Collectively, these cases demonstrate that direct imaging can bridge pharmacology and clinical decision-making by identifying existing tracers suitable for pre-therapeutic decision-making in high-cost, high-toxicity treatments with available alternatives. While nearly half of all drug targets can already be visualized directly, many remain inaccessible to current imaging agents and thus require indirect approaches based on co-expression networks.

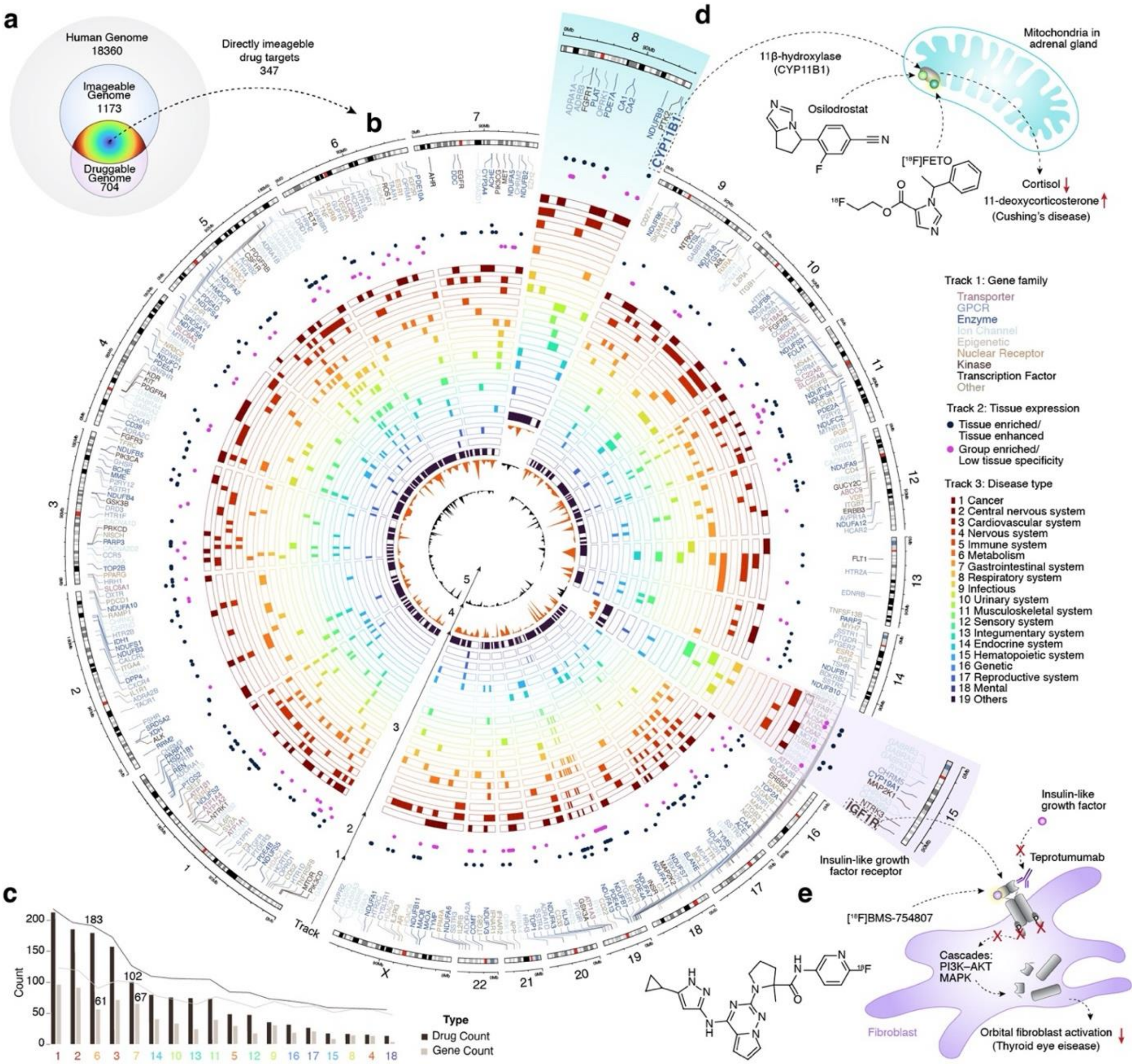

Figure 3. Functional landscape of 347 directly imageable druggable genes. a, Overlap of the imageable and Druggable Genome within the human genome (GRCh38). The number of protein-coding genes is shown for each category. **b**, Circos plot of genomic and functional annotation of 347 directly imageable druggable genes. **Track 1:** chromosomal distribution; **Track 2:** Tissue-specific expression; **Track 3:** Disease classification; **Track 4:** Red histograms show the number of imaging tracers per gene. **Track 5:** Black histograms show the number of approved drugs per gene. **c,** Number of approved drugs and directly imageable druggable genes per disease category. **d**, Osilodrostat blocks 11β-hydroxylase catalytic site in treating Cushing's disease. The expression level of 11β-hydroxylase is visualized with imaging tracer [^{18}F]FETO. **e**, Teprotumumab targets insulin-like growth factor-1 receptor (IGF-1R) in thyroid eye disease. The drug binding caused IGF-1R degradation is visualized with imaging tracer [^{18}F]BMS-754807. Chemical structures of drugs and imaging tracers are shown in each panel.

Reviewer #5 (Remarks to the Author):

I have been asked to review the revised manuscript and have not seen the previous version so apologies if some of my comments may have been made before.

Comment 1:

The title seems to be far too general. This is not about the total druggable genome, but more about the repurposing part. This should be made clear from the start to prevent unnecessary confusion.

Response:
We thank the reviewer for this important observation and agree that clarity of scope is essential. The primary aim of this study is to establish a systematic framework linking approved drugs, their molecular targets, and corresponding molecular imaging probes at genome scale.

To better reflect this scope, we have revised the title to:

"Imaging the Druggable Genome: Linking Approved Drug Targets to Molecular Imaging."

This revised title clarifies that the manuscript focuses on mapping clinically actionable drug–target relationships to molecular imaging strategies, rather than implying coverage of the entire theoretical druggable genome. While the framework enables identification of opportunities to repurpose existing imaging tracers, we consider this a downstream application of the broader integrative mapping rather than the central defining feature of the study.

We believe the revised title now accurately captures both the scope and the translational intent of the work.

Comment 2:

Why did the authors come up with their own definition of the druggable genome using bespoke linkages and how does this compare against relatively canonical definitions provided by others such a opentargets.org?

Response:
We thank the reviewer for this important point and agree that alignment with established terminology is essential.

In this study, we do not introduce a new definition of the druggable genome. Instead, we focus on a widely recognized subset, namely the **currently drugged portion of the druggable genome**, defined as genes encoding targets of FDA- and EMA-approved drugs. This distinction is commonly made in the literature to differentiate between the broader set of potentially druggable genes (estimated at ~4,000) and those that are already clinically targeted.

Our rationale for focusing on this subset is to ensure immediate translational relevance. By restricting the analysis to approved-drug targets, we enable direct linkage between existing therapeutics, molecular imaging probes, and potential companion diagnostic strategies.

In contrast, resources such as OpenTargets include a broader landscape of predicted and investigational targets. While highly valuable for target discovery, these extend beyond the scope of this study, which is designed to map clinically actionable drug–target–imaging relationships.

To clarify this point, we have revised the manuscript throughout to explicitly refer to the **currently drugged portion of the druggable genome**, and we have added text in the Introduction and Discussion to position our framework as complementary to broader resources such as OpenTargets. Please find the revised sentences in Discussion section below:

“The present study provides a comprehensive framework to visualize the currently drugged portion of the Druggable Genome in vivo, establishing a direct link between pharmacology and molecular imaging. In this study, we define the currently drugged portion of the Druggable Genome as genes encoding targets of FDA-/EMA- or country-level approved drugs, prioritizing immediate translational relevance. This clinically grounded approach enables direct linkage between existing therapeutics and imaging strategies for patient stratification and treatment monitoring. In contrast, broader definitions (e.g., OpenTargets) include predicted or investigational targets that expand the target landscape but fall outside the scope of this work. Our framework is therefore complementary, providing a focused, actionable subset that can be extended in future studies to incorporate emerging targets. Importantly, this work systematically maps the FDA-/EMA- or country-level-approved Druggable Genome to currently available molecular imaging tracers through both direct target imaging and indirect imaging via co-expressed imageable genes in disease-relevant tissues. The resulting drug–target–tracer relationships, including family classifications, direct imageable targets, and disease-specific indirect imaging networks across neurology, cardiology, and oncology, are provided in **Supplementary Data 1–6**. These curated source tables establish a reusable translational resource linking pharmacology, transcriptomics, and molecular imaging across clinically actionable disease contexts.”

Comment 3:

Despite the authors claiming to have developed a resource nothing is made publicly available and they simply ask people to contact the lead author. Possibly I am mistaken and everything is actually shared? Otherwise what is the point of the paper?

Response:

We thank the reviewer for raising this important point and apologize for the lack of clarity in the previous version.

In the revised manuscript, we have ensured that the core outputs of this study are provided as **Supplementary Data**, enabling full transparency and reproducibility. Specifically, we provide datasets that systematically link:

- all FDA- and EMA-approved drugs to their molecular targets,
- these drug targets to one or multiple molecular imaging targets, and
- these imaging targets to one or multiple corresponding imaging tracers.

This integrated mapping establishes, for the first time, a comprehensive lineage between approved drugs and potential companion molecular imaging strategies.

All underlying datasets are derived from publicly available sources (Pharos, ChEMBL, DrugCentral, GTEx, TCGA, GEO, MICAD, etc.), and the processed mappings generated in this study are provided in the Supplementary Data files to allow direct reuse and validation by the community.

We have revised the Data Availability section accordingly to clearly reflect that all relevant data required to reproduce the analyses and explore the drug–target–imaging relationships are publicly accessible within the manuscript and its Supplementary Data:

DATA AVAILABILITY

All datasets analysed in this study are publicly available. Drug, target, disease and ligand activity were retrieved from Pharos, ChEMBL, DrugCentral, and the Human Disease Ontology. Imaging target information was obtained from Molecular Imaging and Contrast Agent Database (MICAD), PubMed, and DisGeNET. Drug approval year information was sourced from Drugs@FDA.

Transcriptomic data from normal tissues were sourced from the Genotype-Tissue Expression (GTEx) project (https://gtexportal.org). The oncology results published here are in part based upon data generated by The Cancer Genome Atlas (TCGA) Research Network: https://www.cancer.gov/tcga, the Therapeutically Applicable Research to Generate Effective Treatments (https://www.cancer.gov/ccg/research/genome-sequencing/target) initiative, phs000471. The data used for this analysis are available at the Genomic Data Commons (https://portal.gdc.cancer.gov). Disease-specific transcriptomic datasets were sourced from the following datasets: TCGA cohorts

including TCGA-THCA, TCGA-PAAD, TCGA-MESO, TCGA-SARC, TCGA-PRAD, TCGA-LUSC, TCGA-LUAD, TCGA-KIRC, TCGA-KICH, TCGA-LIHC, TCGA- GBM, TCGA-COAD, TCGA-STAD, TCGA-OV; the ARCHS4 data repository; Gene Expression Omnibus (GEO; https://www.ncbi.nlm.nih.gov/geo/): including GSE140393 (epilepsy), GSE117261 (pulmonary arterial hypertension), and GSE177522 (thymus); and the Therapeutically Applicable Research to Generate Effective Treatments (TARGET) program. TARGET-WT (Wilms Tumor) dataset. National Cancer Institute. Available at: https://portal.gdc.cancer.gov/projects/TARGET-WT 73.

For retrospective survival analyses, genomic and clinical data from docetaxel-treated Breast Invasive Carcinoma (TCGA PanCancer Atlas) cohort available through cBioPortal. Data are provided in Supplementary Data 10 to ensure full reproducibility.

Radiotracer information was compiled from the Molecular Imaging and Contrast Agent Database (MICAD) and peer-reviewed literature. Gene co-expression matrices and full list of drug–target–tracer mappings generated during this study are available from the corresponding author upon reasonable request.

The source data for Figure 2b-d is in Supplementary Data 11. The source data for Figure 3b-c is in Supplementary Data 12. The source data for Figure 6d-e is in Supplementary Data 13.

Comment 4:

I am glad to see the authors have taken on the comments from reviewer's 3 and 4 - the response seems fine. next time please copy the changed section verbatim in the rebuttal letter, makes it easier to say yes.

Response:

We thank the reviewer for this helpful suggestion and we fully agree with this comment. In this revision, we have ensured that key modified sections are clearly described and copied the changed section verbatim into the rebuttal letter to facilitate assessment.

Reference

1. Sims, J.R.*, et al.* Donanemab in Early Symptomatic Alzheimer Disease: The TRAILBLAZER-ALZ 2 Randomized Clinical Trial. *JAMA* **330**, 512-527 (2023).
2. Pivonello, R.*, et al.* Efficacy and safety of osilodrostat in patients with Cushing's disease (LINC 3): a multicentre phase III study with a double-blind, randomised withdrawal phase. *Lancet Diabetes Endocrinol* **8**, 748-761 (2020).
3. Li, L. & Zhao, W. Safety assessment of Osilodrostat: The adverse event analysis based on FAERS database by means of disproportionality analysis. *PLoS One* **20**, e0329088 (2025).
4. Pivonello, R., De Leo, M., Cozzolino, A. & Colao, A. The Treatment of Cushing's Disease. *Endocr Rev* **36**, 385-486 (2015).
5. Wadsak, W.*, et al.* [18F]FETO for adrenocortical PET imaging: a pilot study in healthy volunteers. *European Journal of Nuclear Medicine and Molecular Imaging* **33**, 669-672 (2006).
6. Chen, T. & Tao, J. Nonsurgical Management of Thyroid Eye Disease: Efficacy, Options, and Costs. *Ophthalmic Pearls* (2025).
7. Xu, Z.*, et al.* Teprotumumab in the management of thyroid eye disease mechanistic insights and adverse reactions: a comprehensive review. *Front Endocrinol (Lausanne)* **16**, 1480195 (2025).
8. Li, X.*, et al.* Recent advances in graves ophthalmopathy medical therapy: a comprehensive literature review. *Int Ophthalmol* **43**, 1437-1449 (2023).
9. Majo, V.J.*, et al.* Synthesis and in vitro evaluation of [18F]BMS-754807: a potential PET ligand for IGF-1R. *Bioorg Med Chem Lett* **23**, 4191-4194 (2013).
