# Supplementary material for: Repurposing molecular imaging to map drug targets in vivo
Source: Commun Med (Lond). 2026 Jul 13;6:579. doi: 10.1038/s43856-026-01749-6 (PMC13745577; doi:10.1038/s43856-026-01749-6)
Supplement: Supplementary file 2 [file 43856_2026_1749_MOESM2_ESM.pdf]

Supplementary Information

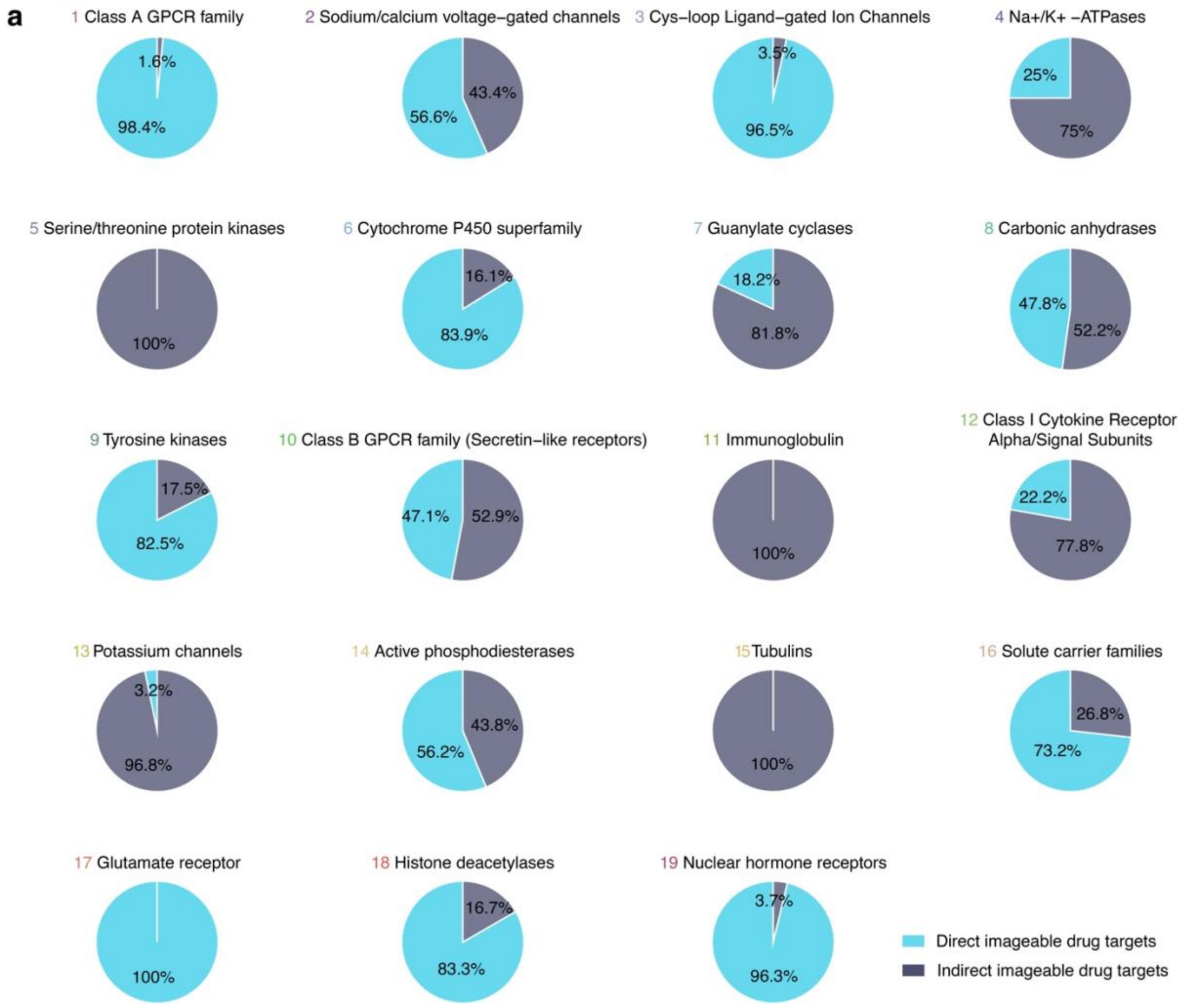

Supplementary Figure 1. Imaging coverage across 19 major gene families. Each pie chart shows that, for a gene family, the proportion of directly imageable genes versus indirectly imageable genes.

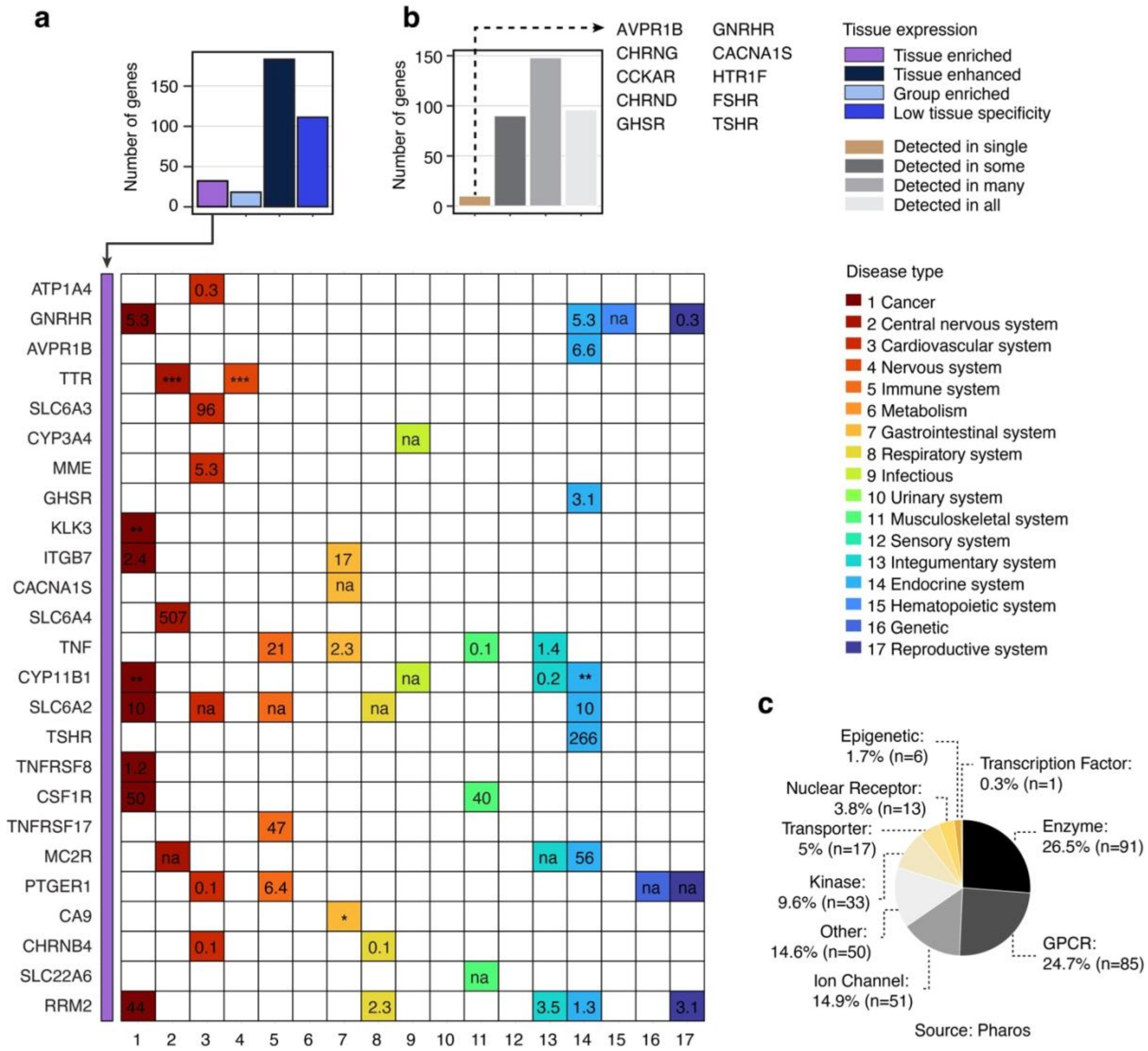

Supplementary Figure 2. Tissue-specific expression levels by disease category. a, the Human Protein Atlas (HPA) assessment on tissue-specific expression (Tissue expression specificity) and **b**, detectable baseline expression across tissues (Tissue distribution). Tissue enriched >= 4-fold higher mRNA in a given tissue compared to any other; Group enriched >=4 fold higher average mRNA in 2-5 tissue compared to any other; Tissue enhanced >=4 fold higher mRNA level in a given tissue compared to average of all other tissues; Detected in single: detected in a single tissue; Detected in some: detected in more than one but less than 1/3 of tissues; Detected in many: detected in at least 1/3 but not all tissues. Heatmap: Color-coded columns represent disease groups. Values are average normalized transcripts per million (nTPM) from relevant tissue RNA sequencing data retrieved from the Human Protein Atlas. Significance: * >100 nTPM, ** >1000 nTPM, *** >10,000 nTPM; "na" indicates no detectable expression. **c**, Protein class annotation for all 343 direct imageable druggable genes and d, their associated FDA drug mechanism of action.

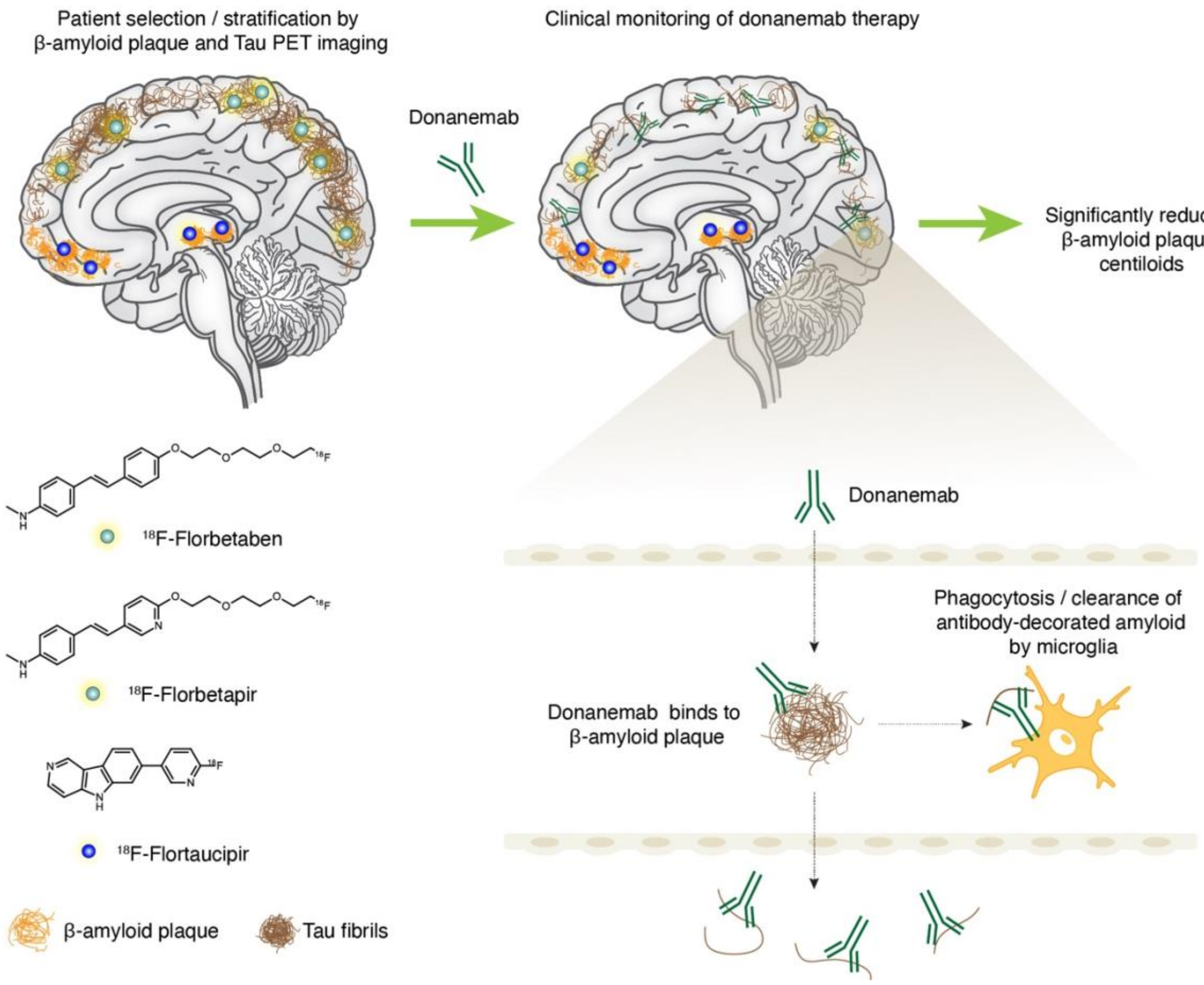

Supplementary Figure 3. Imaging-guided patient enrichment and treatment monitoring for donanemab in early symptomatic Alzheimer's disease. Participants undergo PET confirmation of β-amyloid plaque positivity using amyloid PET and are grouped by tau enrichment using tau PET, then receive donanemab. Serial amyloid PET is used to quantify treatment-associated amyloid plaque reduction.

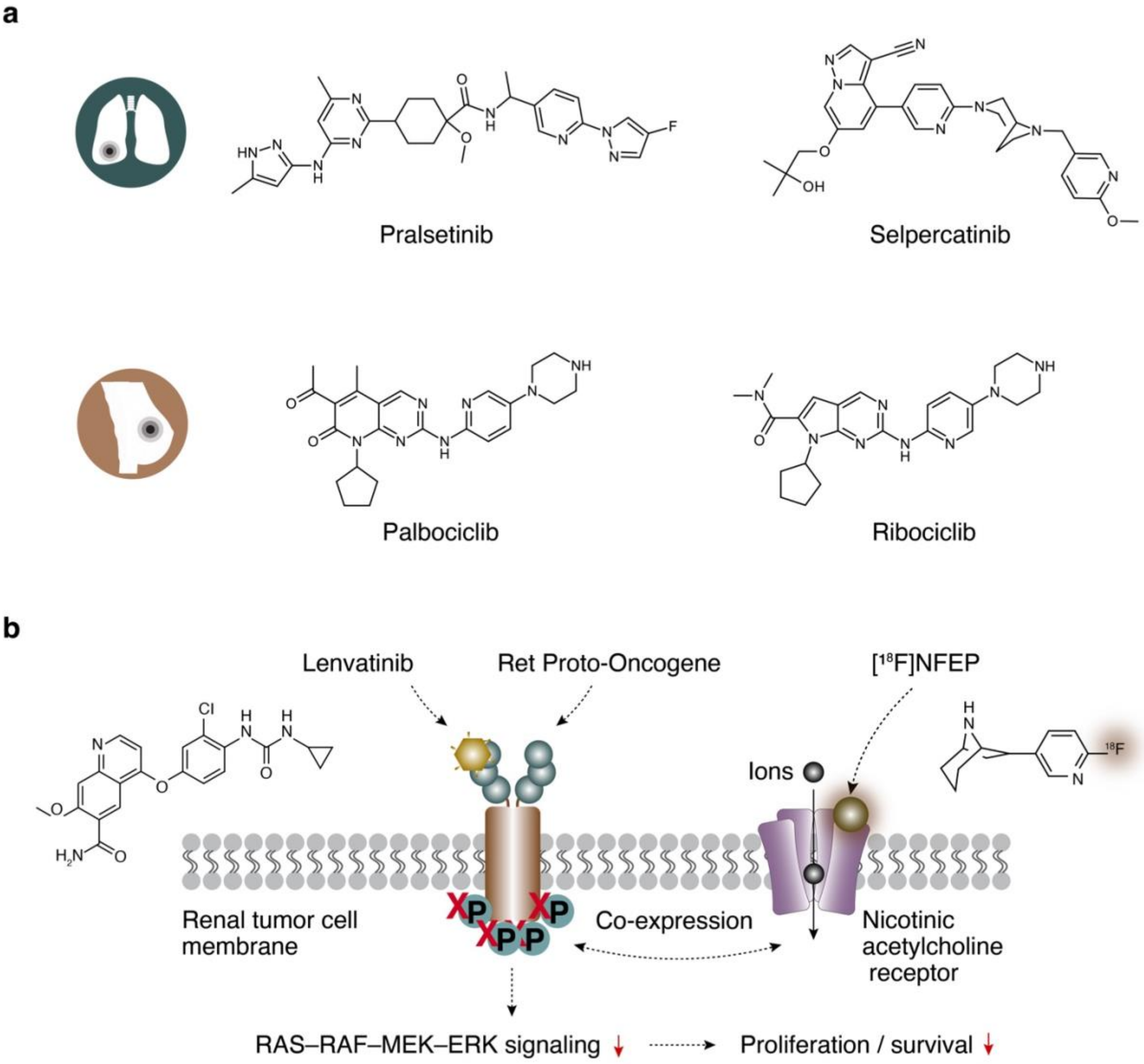

Supplementary Figure 4. Drugs and imaging concept. a, Chemical structures of selected anti-cancer drugs treating lung and breast cancer. **b,** An illustration of an imaging target, nicotinic acetylcholine receptor, as a potential companion diagnostic target for lenvatinib treatment in advanced renal cell carcinoma.

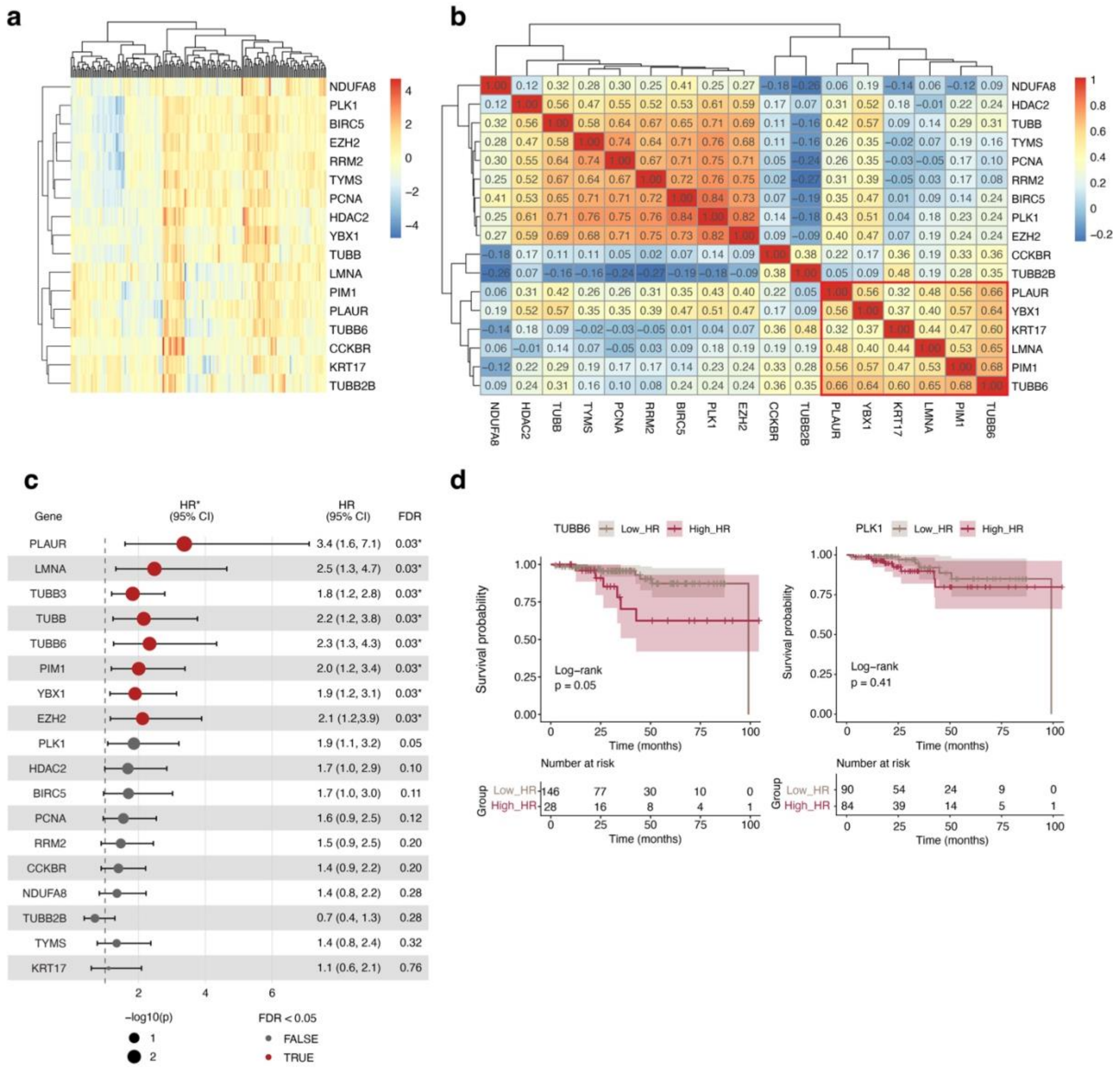

Supplementary Figure 5. Docetaxel target, TUBB6, coexpression landscape and prognostic value in invasive breast carcinoma. a, Heatmap of normalized expression of two docetaxel target genes, TUBB and TUBB6, and 3 co-expressed genes among 175 breast invasive carcinoma patients treated with docetaxel. **b**, Heatmap of spearman's correlation score (r) between PIM1, TUBB6 and other 3 genes among 175 invasive breast carcinoma patients treated with docetaxel (FDR<0.01). **c**, Stage-adjusted Cox Forest plot of drug target gene and their co-expressed genes (1102 TCGA breast invasive carcinoma patients, r >0.5, p value <2.0e-70). **d**, Cox regression model–based survival curves grouped by high HR (TUBB6: >2.3; PLK1: >1.1) vs low HR (TUBB6: ≤2.3; PLK1: ≤1.1) in docetaxel treated breast invasive carcinoma patients. Shaded areas show 95% CIs. HR: Hazard ratios; CI, confidence interval.
