# Supplementary material for: Repurposing molecular imaging to map drug targets in vivo
Source: Commun Med (Lond). 2026 Jul 13;6:579. doi: 10.1038/s43856-026-01749-6 (PMC13745577; doi:10.1038/s43856-026-01749-6)
Supplement: Supplementary file 3 [file 43856_2026_1749_MOESM3_ESM.docx]

Supplementary Data 1: Drug, target and protein functional classification of 704 drug target genes.

Supplementary Data 2: Drug approval information.

Supplementary Data 3: Summary of direct and indirect imageable targets.

Supplementary Data 4: Tracers indirectly imaging the neurological disease drug targets.

Supplementary Data 5: Tracers indirectly imaging the cardiovascular disease drug targets.

Supplementary Data 6: Tracers indirectly imaging the oncological disease drug targets.

Supplementary Data 7: Full list of imageable genes positively co-expressed with indirect imageable drug targets in epilepsy patients (GEO study: GSE140393).

Supplementary Data 8: Full list of imageable genes positively co-expressed with indirect imageable drug targets in pulmonary arterial hypertension patients (GEO study: GSE117261).

Supplementary Data 9: Full list of imageable genes positively co-expressed with indirect imageable drug targets in TCGA renal cell carcinoma patients.

Supplementary Data 10: Study de-identifiers downloaded from cBioPortal, docetaxel-treated TCGA-BRCA cohort.

Supplementary Data 11: Source data for Figure 2

Supplementary Data 12: Source data for Figure 3

Supplementary Data 13: Source data for Figure 6
